# Single‐Cell Transcriptomic Atlases of Camels and Cattle Unravel Molecular Evolution of Digestive and Metabolic Systems

**DOI:** 10.1002/advs.202519346

**Published:** 2026-02-03

**Authors:** Tao Shi, Huiquan Shan, Haoping Wang, Xi Guo, Houcheng Li, Bo Han, Senlin Zhu, Fei Wang, Guanghui Tan, Zhannur Niyazbekova, Jilong Ren, Yaqi Zhou, Qi Zhang, Weijie Zheng, Minghui Jia, Ao Zhang, Xuesha Cao, Huizeng Sun, Dongxiao Sun, Lingzhao Fang, Yi Zheng, Xihong Wang, Yu Jiang

**Affiliations:** ^1^ Key Laboratory of Animal Genetics Breeding and Reproduction of Shaanxi Province College of Animal Science and Technology Northwest A&F University Yangling China; ^2^ State Key Laboratory of Molecular Biology Center For Excellence in Molecular Cell Science Shanghai Institute of Biochemistry and Cell Biology Chinese Academy of Sciences Shanghai China; ^3^ Center for Quantitative Genetics and Genomics Aarhus University Aarhus Denmark; ^4^ Key Laboratory of Animal Genetics National Engineering Laboratory For Animal Breeding Department of Animal Genetics College of Animal Science and Technology Breeding and Reproduction Breeding and Reproduction of Ministry of Agriculture and Rural Affairs China Agricultural University Beijing China; ^5^ Institute of Dairy Science College of Animal Sciences Ministry of Education Key Laboratory of Molecular Animal Nutrition Zhejiang University Hangzhou China; ^6^ Reference center For Safety and Quality of Agricultural Products Kazakh National Agrarian Research University Almaty Kazakhstan

**Keywords:** camel, cattle, kidney, multi‐chambered stomach evolution, single‐cell transcriptomic atlas

## Abstract

Cellular and molecular characterization of mammals with multi‐chambered stomachs is crucial to our understanding of evolution in digestive and metabolic systems. Here, we generate single‐cell transcriptomic atlases of 253,448 and 279,057 cells from 54 tissues in camels and cattle, discovering 124 cell types. Cross‐species comparisons at four developmental stages reveal a common evolutionary origin among camel glandular sac, third‐chambered stomach, and bovine abomasum, with the camel lineages uniquely harboring a cell population characterized by heightened expression of genes involved in cell proliferation. Also, the advantages of camel hepatocytes in prevention of excessive fat deposition are uncovered. Interestingly, the spatial transcriptomic analysis further reveals a unique population of *S100A4^+^* vascular smooth muscle cells in camel rather than other mammalian kidneys, potentially involved in prevention of renal hypertension. Altogether, our study provides invaluable resources for evolutionary biology and highlights cellular innovations underlying mammalian adaptation.

## Graphical abstract

We generated multi‐tissue single‐cell transcriptomic atlases of camels and cattle, uncovering conserved and lineage‐specific cellular features across digestive and metabolic systems. Cross‐species comparisons revealed the evolutionary origin of the camel glandular sac and identified novel cell populations linked to physiological specialization, providing new insights into the evolution of multi‐chambered mammals at single‐cell resolution.

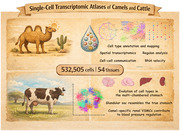

## 1 Introduction

*Cetartiodactyla* encompasses several multi‐chambered stomach animal species that share a common ancestor approximately 64 million years ago and that display remarkable evolutionary differences to adapt to diverse habitats, including grasslands, deserts, and oceans [1, 2]. The four‐chambered stomach system of ruminants has been thoroughly studied, but the multi‐chambered stomachs of camels and whales have been rarely investigated. As a pseudo‐ruminant, the camel serves as both an ideal comparative model for dissecting the biology of true ruminants and an underexplored evolutionary reservoir of its own adaptive innovations. Camels have evolved a unique distributed digestion strategy: the GS (glandular sac) in their fore‐stomach region secretes digestive fluids [3, 4], initiating enzymatic hydrolysis earlier and extending the overall digestion time [5, 6]. This sharply contrasts with the “centralized digestion” mode of true ruminants, in which enzyme secretion is strictly limited to the final stomach chamber, highlighting the evolutionary divergence of their digestive systems. Nevertheless, the biological significance of the camel multi‐chambered stomach has not been adequately recognized, and the evolutionary history of the GS, as well as the specific cell types responsible for enzyme secretion, remain to be elucidated.

The different sources of food and environmental stress also shape the species‐specific metabolic organs. Camels in physiological states exhibit high insulin resistance, robust fat storage and conversion capabilities, and resistance to hypertension [7], which provides implications for treatment of human disorders such as obesity, fatty liver, and hypertension [8, 9]. To date, the comparative analysis of multi‐chambered animals has been conducted at the genomic level [1, 3], but the key cellular and molecular mechanisms driving the unique traits of multi‐chambered animals remain largely illusive. Specifically, previous cross‐species comparative single‐cell transcriptomic studies have been confined to several tissue types in ruminants [10, 11, 12], and even no single‐cell transcriptomic study has been conducted in camels yet. In this sense, generation of comprehensive multi‐organ single‐cell transcriptomic atlases in camels and cattle is essential for identification of specialized cell lineages across tissues between species.

Here, to systematically elucidate the cellular basis underlying the adaptive traits of multi‐chambered animals, we constructed multi‐tissue single‐cell transcriptomic atlases for camels and cattle. These two species represent distinct evolutionary lineages within multi‐chambered mammals and display pronounced differences in digestive physiology, metabolic regulation, and environmental resilience, making them ideal models for cross‐species comparisons. To comprehensively capture the cellular diversity across major organ systems, we profiled 54 tissues encompassing key metabolic, digestive, and circulatory systems, thereby generating a foundational dataset that enables cross‐species analyses at single‐cell resolution. The overarching goals of this study were to characterize species‐specific differences in cellular composition and molecular features within digestive and metabolic systems, to elucidate the cellular basis of adaptive evolution in multi‐chambered animals, and to provide a valuable framework for future research in evolutionary biology.

## 2 Results

### 2.1 Single‐Cell Transcriptomic Atlases of Camels and Cattle

**FIGURE 1:**
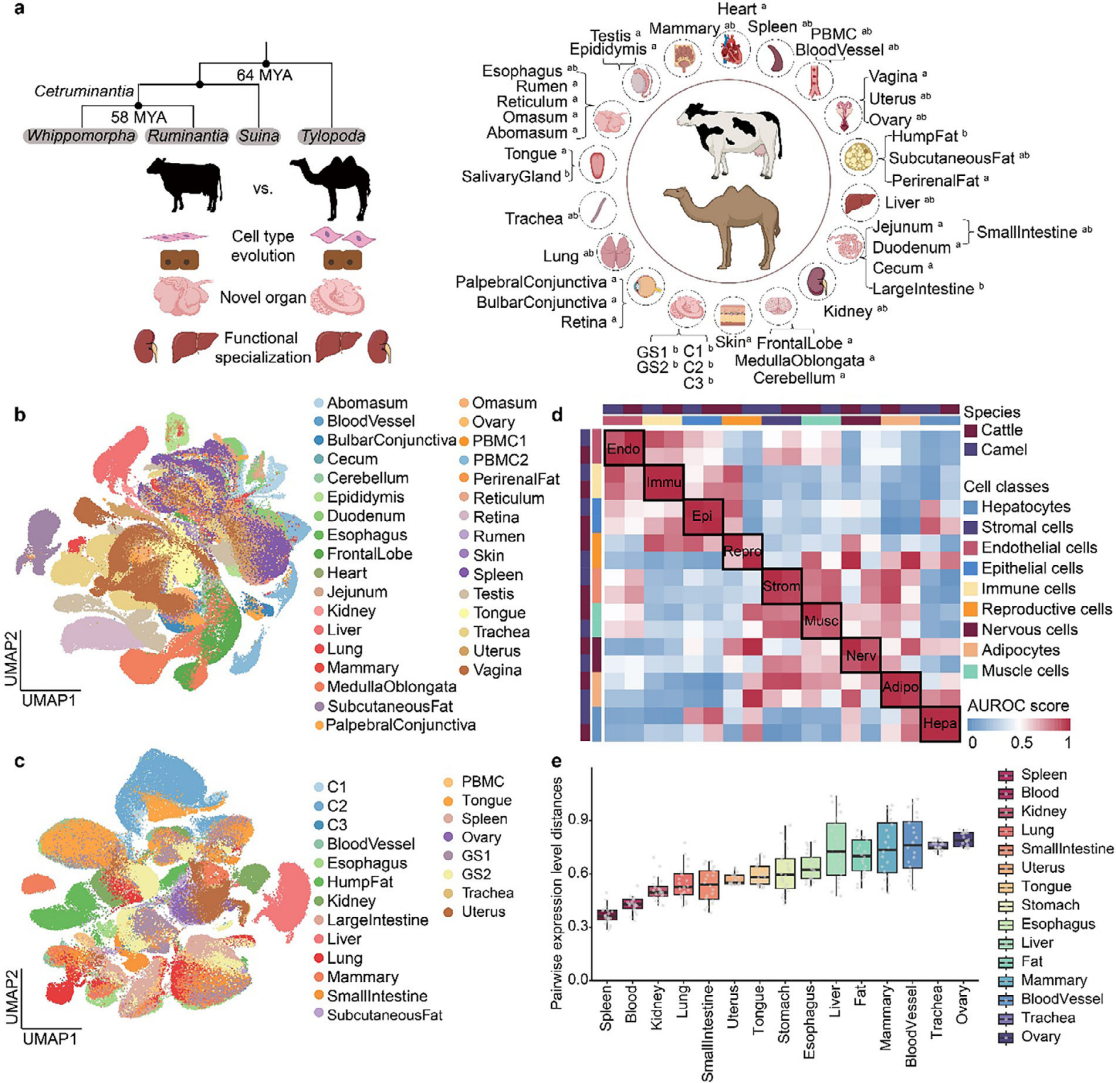
The single‐cell transcriptomic atlases of tissues from camels and cattle. a, A phylogenetic tree of *Artiodactyla* (left), highlighting the phylogenetic position and the diverse digestive systems of camels and cattle. The schematic diagram (right) displays the tissues analyzed in this study, and superscripts “a” and “b” represent the tissues from cattle and camels, respectively. b, c, The Uniform Manifold Approximation and Projection (UMAP) plots showing the global clustering of all cells from the dataset colored by tissues (n  =  264,472 individual cells/nuclei in cattle and n  =  160,682 in camels). d, Correlation of orthologous gene expression between cell classes from adult cattle and camels. The Spearman's correlation coefficient calculated by AUROC scores was used to measure the similarity of cell classes: red, high correlation; blue, low correlation. The cell classes and species are marked by different colors. e, The camel‐cattle pairwise comparison of tissues (n = 15). 1‐rho, 1‐Spearman's correlation coefficient in the vertical axis represents heterogeneity in tissues between species. PBMC, peripheral blood mononuclear cell; C1, C2, and C3 represent the first‐, second‐, and third‐ chambered stomach in camels, respectively; GS1 and GS2 represent anterior and posterior glandular sac areas, respectively.

Our study generated single‐cell RNA‐sequencing (scRNA‐seq) or single‐nucleus RNA‐sequencing (snRNA‐seq) data from 21 adult camel tissues, comprising 160,682 cells, and from 33 adult bovine tissues, comprising 264,472 cells (Table S1). Based on the expression of canonical markers, we annotated 124 cell types, representing 78 and 106 cell types in camels and cattle, respectively. Among these cell types, 59 were shared between adult camels and cattle (Figures 1a–c; Tables S2 and S3), and each tissue contained an average of approximately 13 distinct cell types. Cell types identified in adult individuals were grouped into ten major cell classes, including adipocytes (n = 13,111, making up 3.08% of all cells in the adult individual), endothelial cells (n = 60,490, 14.23%), epithelial cells (n = 63,895, 15.03%), immune cells (n = 142,965, 33.59%), muscle cells (n = 24,547, 5.77%), neuronal cells (n = 38,967, 9.21%), hepatocytes (n = 20,024, 4.71%), germ cells (n = 5,637, 1.32%), stromal cells (n = 48,129, 11.32%), and unassigned cells (n = 7,389, 1.74%) (Table S4). The unassigned cells showed high expression levels of ribosomal (*RPL6* and *RPS15*) and histone genes (*H2AC14* and *H2BC18*), suggesting an intermediate state during cell transformation or cell cycle. The endothelial cells (*PECAM1* ^+^) were shared among 20 camel and 30 bovine tissues, while fibroblasts (*DCN* ^+^ and *COL1A1* ^+^) were distributed in 19 camel and 32 bovine tissues.

We then employed the 15,324 one‐to‐one orthologous genes identified by eggNOG [13] to integrate single‐cell transcriptomic data from camels and cattle (Table S5). The MetaNeighbor [14] correlation analysis showed that the same cell classes from the two adult species clustered well. Of all the nine cell classes (except the unassigned cells), hepatocytes, endothelial cells, immune cells, and epithelial cells showed higher similarities between the two species (AUROC > 0.95), suggesting that the similarities in cell classes based on orthologous gene expression prevail over the species differences, in line with previous studies on the conservation of mammalian cell types [15]. Compared to other cell classes, germ cells showed a lower similarity between species, probably because germ cells are the cell type with the most rapid evolutionary rate (Figure 1d) [16]. To quantify the evolutionary conservation and differentiation of tissues across species, we calculated the pairwise gene expression distance based on pseudo‐bulk transcriptomic data. Following established methods [16], we utilized 1 – Spearman correlation coefficient between the pseudo‐bulk profiles of paired tissues from camels and cattle to measure the heterogeneity of their evolutionary gene expression programs. A smaller distance value indicates greater transcriptional conservation. For tissues that could not be directly matched at high resolution, such as the specialized multi‐chambered stomachs, we integrated them into broader tissue categories to ensure a consistent and robust cross‐species comparison. The result showed that spleens and peripheral blood mononuclear cells (PBMCs) exhibited relatively low gene expression distances (paired gene expression distance < 0.5), and both tissues were predominantly composed of immune cells (Figure S1a–c). In contrast, ovaries, among all the tissues, showed the highest gene expression distance between camels and cattle (paired gene expression distance > 0.7) (Figure 1e; Figure S1d). Additionally, we performed a paired comparison for cell classes and tissues between the two species, by using the SAMap pipeline [17]. Except for the higher heterogeneity in cell classes in ovaries, the cell classes in other tissues showed good integration between the two species. Immune cells and hepatocytes remained the most conserved cell classes between two species, having the highest alignment scores (Figure S2a–c), in alignment with the results generated from pseudo‐bulk transcriptomes and MetaNeighbor. Altogether, these results provide a comprehensive overview of the cell type composition of multiple tissues in camels and cattle, characterizing the conservation and heterogeneity of corresponding cell types between the two species.

### 2.2 Heterogeneity of Structural Cells in the Multi‐Chambered Stomachs

**FIGURE 2:**
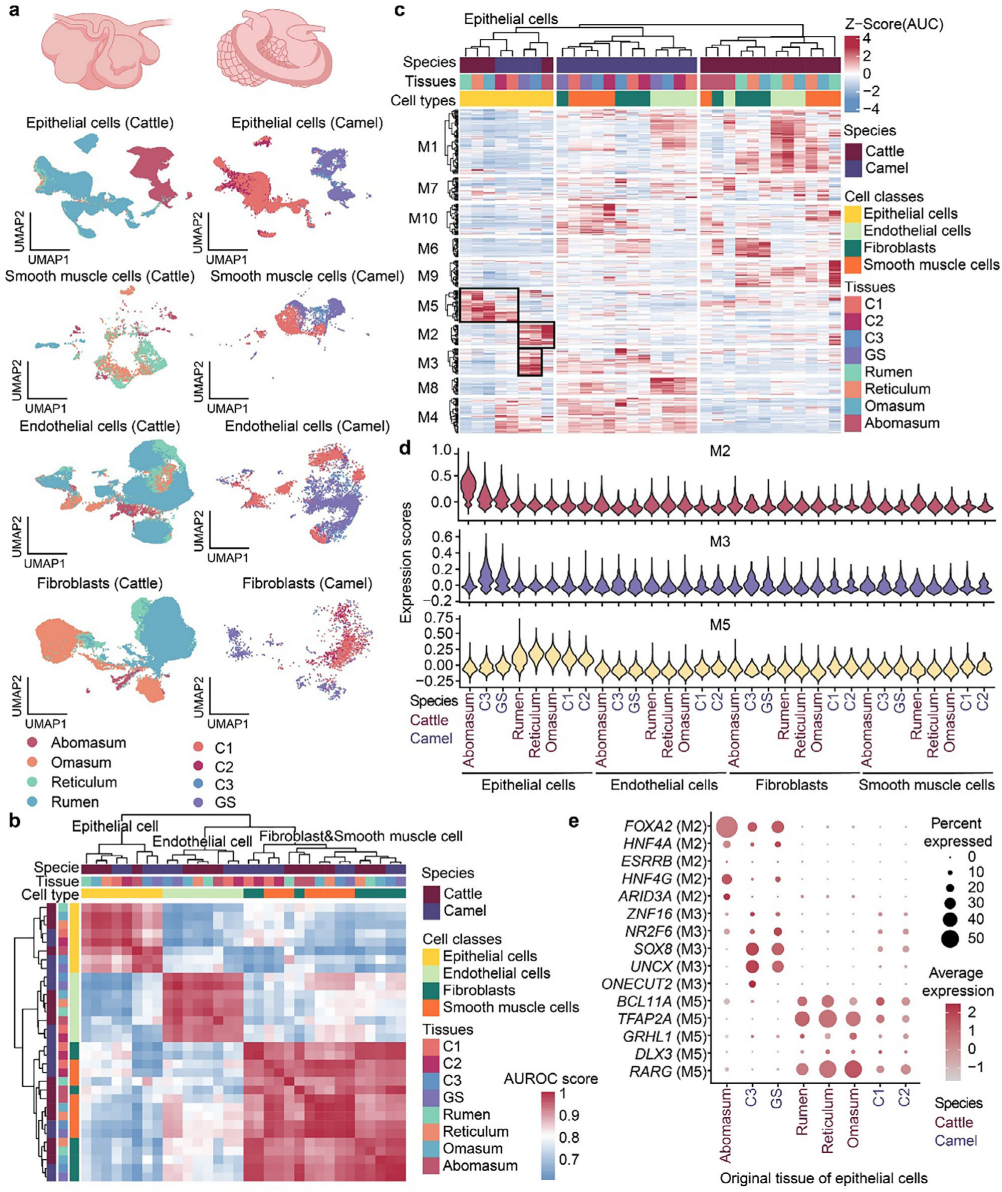
The cross‐species comparison of structural cells in the multi‐chamber stomachs. a, The integration of structural cells from stomach chambers. UMAP plots represent the integration of each structural cell (row) from bovine (left column) and camel (right column) multi‐chambered stomachs. b, Hierarchical clustering of structural cells based on AUROC scores. Different colors of the rows above the heatmap represent different species, stomach chambers, or cell types, and the different colors in the heatmap represent the Spearman's correlation coefficient calculated by AUROC scores. c, The regulon activity scores of structural cells, highlighting the specific modules in epithelial cells (M2, M3, and M5). d, The gene scoring analysis of structural cells, and the gene sets generated by M2, M3, and M5. e, The dot plot showing the expression levels of top five genes with the highest regulon specificity scores (RSS) in M2, M3, and M5.

The multi‐chambered stomachs are a hallmark of the highly specialized digestive tract in camels and cattle [1]. To investigate the conservation and divergence in the composition of cell types and their gene expression patterns in stomach chambers between camels and cattle, we collected single‐cell transcriptomic data from two additional datasets of bovine stomach chambers to enhance the representativeness of the data [10, 18]. This yielded a total of 224,866 structural cells, encompassing the major cell classes (epithelial cells, smooth muscle cells, endothelial cells, and fibroblasts) involved in tissue architecture across species. Following integration and batch‐effect correction, epithelial cells (*EPCAM* ^+^) formed two cell categories based on anatomical regions: cells from the forestomach (n = 79,696) and cells from the true stomach and GS (n = 33,923). Conversely, the remaining structural cells, including endothelial cells (*PECAM1* ^+^; n = 63,267), fibroblasts (*DCN* ^+^; n = 41,935), and smooth muscle cells (SMCs, *ACTA2* ^+^; n = 6,045), clustered primarily by cell types, independent of their specific stomach chambers (Figure 2a). To explore gene regulatory networks across cell types, we subsequently detected 357 regulons and calculated their activity scores using the SCENIC method [19]. Each regulon consists of the transcription factor (TF), its enriched motif, and the set of target genes, serving as the principal basis for identification of homologous relationships between cell types. The hierarchical clustering of regulon activities showed interspecies differences, particularly in the endothelial cells, fibroblast cells, and SMCs, despite similar orthologous gene expression in corresponding cell types between species. The similarity in regulon activities, which was in line with the similarity in orthologous gene expression, outweighed the species differences in epithelial cells, resulting in two clustering patterns: abomasum/C3/GS and rumen/omasum/reticulum/C1/C2 (C1, C2 and C3: first‐, second‐ and third‐chambered stomach) (Figure 2b). This clustering pattern indicates a high level of specialization in the epithelial cells between the true stomach and the forestomach, while other structural cells exhibit greater interspecies plasticity in gene expression patterns indicative of the faster evolutionary rate.

To gain more knowledge about the conservation and innovation of gene regulation patterns in multi‐chambered gastric epithelial cells between the two species, we further clustered these regulons into ten modules (M1‐M10) according to activity scores, and found M2, M3 and M5 specific to epithelial cells in C3/GS/abomasum, C3/GS, and forestomachs, respectively. Intriguingly, M3 was specifically enriched in the epithelial cells of camel GS and C3 but not in those of bovine abomasum, suggesting the species‐specific gene regulation in epithelial cells from camel GS and C3 (Figure 2c; Table S6). The expression levels of gene sets consisting of TFs and genes in M2, M3, and M5 also demonstrated similar species‐ and tissue‐specificity (Figure 2d). Next, the top five regulons with the highest regulon specificity scores (RSS) in M2, M3, and M5 were inspected. In M2, *FOXA2*, *HNF4A*, *ESRRB*, *HNF4G*, and *ARID3A*, which are associated with secretion and chemical digestion functions, displayed markedly high expression levels in epithelial cells from abomasum, C3, and GS, whereas *ZNF16*, *NR2F6*, *SOX8*, *UNCX*, and *ONECUT2* in M3 exhibited substantially high expression in epithelial cells from C3 and GS, of which *ONECUT2*, *SOX8*, and *NR2F6* have been reported as the effectors of TFs in the proliferation and migration of epithelial cells in the digestive tract [20, 21, 22]. This finding suggests that TFs in M3 might mediate direct migration of GS and C3 epithelial cells. In M5, *BCL11A*, *TFAP2A*, *GRHL1*, *DLX3*, and *RARG* showed high expression levels in the epithelial cells from rumen, reticulum, omasum, C1, and C2 (Figure 2e). These results indicate that the epithelium of the multi‐chambered stomachs in both species is composed of two types of epithelial cells, with both being relatively conserved between the two species. Additionally, there were other regulon modules that showed high activity in specific stromal cells. For instance, M4 and M8 were highly enriched in the structural and endothelial cells from the camel multi‐chambered stomachs, respectively. The TFs in these two regulon modules are associated with development, cell apoptosis, and inflammation regulation (e.g., the *EGR* and *BCL* families, and *SP4*) [23, 24, 25]. Notably, M9 showed high activity in the stromal cells from the forestomach and contained TFs that regulate vascular and epithelial morphology (e.g., *FOXJ2* and the *TBX* family). Another TF harbored in M9 was *TBX3*, of which the expression in fibroblasts is associated with early papillogenesis and papillae keratinization in the rumen [26]. However, the camel C1 lacked both M9 activity and papillary structures in its epithelium and stromal cells. These results indicate that, compared to that of other structural cell types, the similarity of epithelial cells is driven by the tissue rather than the species. Albeit relatively conserved between species, epithelial cells in the abomasum, C3, and GS exhibit heterogeneity that is driven by the TFs and by their target genes harbored in M3. Furthermore, the conserved regulons (specifically modules M2, M3, and M5) shared by epithelial cells of the GS and C3 confirm their homology as cell types derived from a common ancestral lineage.

### 2.3 Single‐Cell Transcriptomic Analysis at Developmental Stages Reveals the Homology Between Camel GS and the True Stomach

**FIGURE 3:**
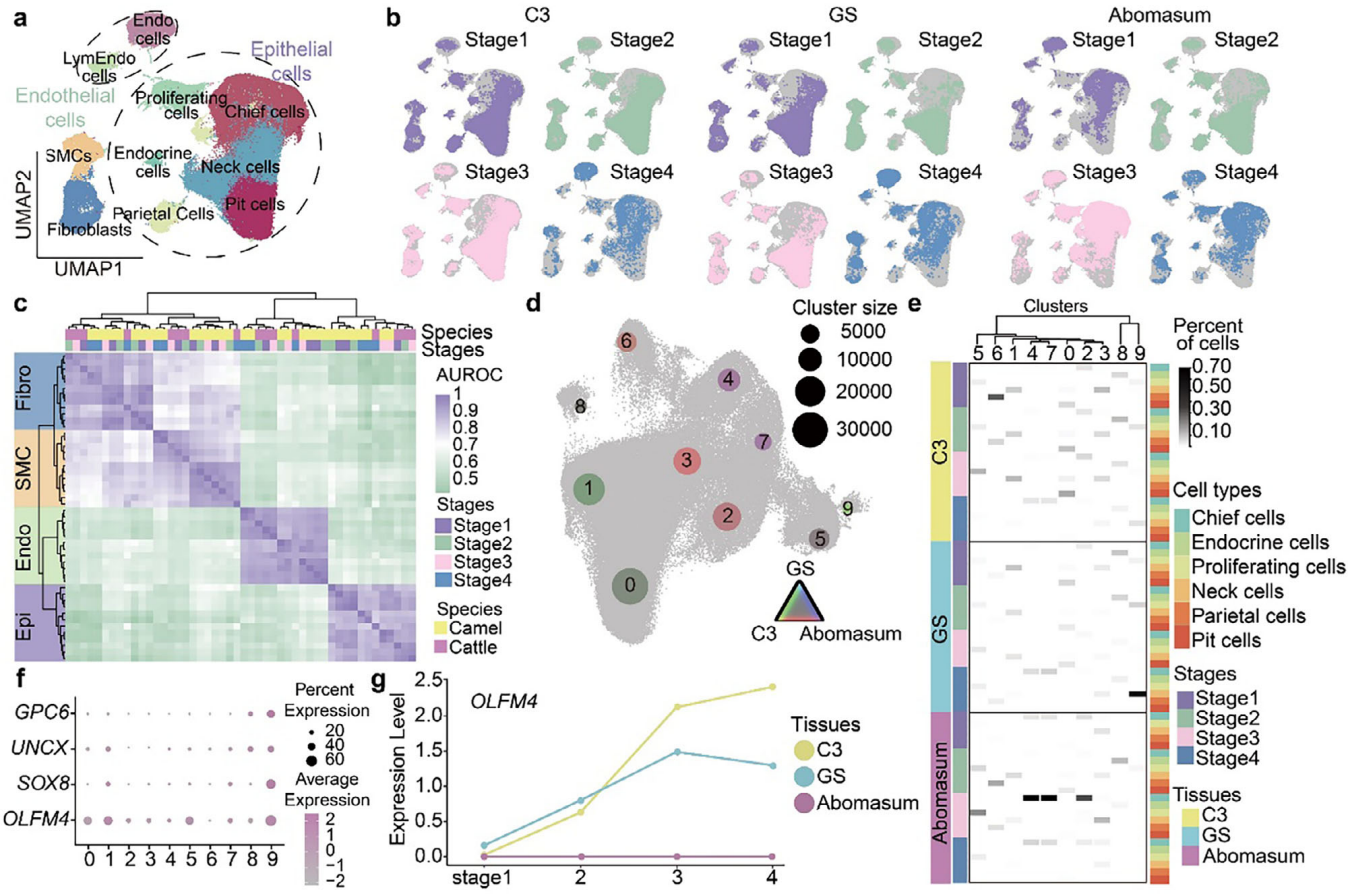
The cross‐species comparison of cell types and gene expression profiles in camel GS/C3 and bovine abomasum at four developmental stages. a, A UMAP plot showing the integration of structural cells from the bovine abomasum and camel GS and C3 at four developmental stages. Epi represents epithelial cells, Endo represents endothelial cells, SMCs represent smooth muscle cells, and LymEndo represents lymphatic endothelial cells. b, UMAP plots of the integrated dataset highlighting cells from each stomach chamber (purple, green, pink, and blue). Stages 1–4 correspond to newborn, pre‐weaning, post‐weaning, and adult, respectively. c, Correlation of orthologous gene expression in structural cells between cattle and camels. AUROC scores were used to assess the similarity of cell classes: purple indicates high correlation, while green and white represent lower correlations. d, A UMAP plot showing the integration of all epithelial cells from the three stomach chambers at four developmental stages. Dot colors represent the tissue composition within each integrated cluster, and dot size reflects the number of cells in each cluster. e, Proportion of epithelial cell types within each integrated cluster. Box colors indicate the degree of tissue mixture (gray represents an equal proportion of cells from each tissue). The dendrogram on the heatmap shows the hierarchical clustering of integrated clusters. f, A dot plot showing the expression levels of selected genes across integrated clusters. g, Developmental stage‐dependent expression of *OLFM4* in epithelial cells from the GS, C3, and abomasum.

To elucidate the developmental origin of the GS and its relationship with the true stomach, we additionally collected and analyzed single‐cell transcriptomic data from the camel GS and C3, as well as the bovine abomasum, across three non‐adult stages (Stages 1–3: newborn, pre‐weaning, and post‐weaning). In total, we obtained 92,766 cells from non‐adult camels and 14,585 cells from non‐adult cattle. These data, together with the adult samples (Stage 4), enabled us to characterize the transcriptomic transitions throughout gastric development. Integrated data from these stages yielded 137,860 structural cells that were further categorized into ten cell types and grouped into four classes: epithelial cells (including six cell types), endothelial cells (two cell types), SMCs, and fibroblasts (Figure 3a). In the GS of newborn and pre‐weaning camels, the expression of epithelial markers, such as *ATP4A* (a parietal cell marker associated with gastric acid synthesis), *PGA5* (a chief cell marker linked to digestive enzyme precursors), and *MUC5AC* (a neck cell marker associated with mucus production [27]), indicates that the GS has already begun to exhibit true stomach‐like functions during early life (Figure S3a,b). The cross‐species similarity analysis at the cell class level and integration analyses revealed that fibroblasts, SMCs, and the two endothelial cell types from the GS, C3, and abomasum consistently co‐clustered by cell classes at all developmental stages. Among epithelial cells, the majority of cell types exhibited consistent co‐clustering patterns, except for certain neck cells and proliferating cells, which showed incomplete co‐clustering across species (Figures 3b,c). These cross‐species similarity analyses revealed the overall resemblance in cellular composition between the GS and the true stomach, and also the epithelial cell diversity within the GS and C3.

Epithelial cells are directly responsible for chemical digestion in the true stomach [28]. To characterize the correspondence among epithelial cell types across the three stomach chambers, we extracted 107,876 epithelial cells and regenerated ten integrated clusters (Figure 3d). We observed that cells predominantly clustered according to lineage specificity. For example, parietal and endocrine cells were enriched in clusters 6 and 8, respectively. Chief cells were mainly found in clusters 2, 4, and 7. Neck and proliferating cells were distributed across clusters 0, 1, 3, and 5. These clusters contained cells from all three stomach chambers across all developmental stages and were organized primarily by lineage identity rather than by species of origin or developmental time points. This conserved clustering pattern indicates shared and lineage‐defining gene expression programs across diverse anatomical and developmental contexts, suggesting that these epithelial lineages originate from primordial gastric epithelial cells and that their fundamental digestive functions have largely been maintained during the evolution of the *Artiodactyla* stomach.

In contrast, cluster 9 was specifically enriched with neck and proliferating cells from the camel GS and C3, representing a camel‐specific epithelial cell subtype (Figure 3e). These cells in cluster 9 highlighted the potential cell‐type innovation in epithelial cell renewal and mucus secretion during the evolutionary development of the GS and C3. Subsequent analysis revealed that *GPC6*, *SOX8*, *OLFM4*, and *UNCX* were specifically upregulated in cluster 9 compared to other epithelial cell clusters (Figure 3f). All four TFs were one‐to‐one orthologs between camels and cattle, with *SOX8* and *UNCX* being members of the camel‐specific regulon module M3 and appearing to drive the epithelial heterogeneity between the camel GS/C3 and the bovine abomasum. These two TFs were expressed in the GS and C3 of camels at all four developmental stages, with their expression peaking at the adult stage. Additionally, *OLFM4*, in particular, is a known marker of gastric cancer cells and intestinal metaplasia reported to promote cell proliferation and regulate cell adhesion and migration [29, 30]. In the GS and C3, *OLFM4* was continuously upregulated during development and its expression stabilized at the post‐weaning and adult stages (Figure 3g). *GPC6*, previously reported to facilitate gastric growth during early life [31], also showed elevated expression in cluster 9 (Figure S3c).

The GS and C3, albeit functionally similar compared to other stomach chambers, differ in morphology and anatomical locations. To delve into the distinctions between these two chambers, we performed paired comparisons of structural cell types in adult camel GS and C3. To minimize false‐positive results, only cell clusters containing 200 or more cells were included in the differential gene expression analysis between matched cell types from the two tissues. Notably, differentially expressed genes (DEGs) in chief cells between the GS and C3 were enriched with Gene Ontology (GO) terms related to animal organ morphology and extracellular space, whereas those in fibroblasts were enriched with GO terms associated with the regulation of cell proliferation and differentiation (Figure S4a–d and Tables S7 and S8). These gene expression differences may ultimately contribute to the unique honeycomb epithelial structure of the GS and support the maintenance of its epithelial secretory functions.

In summary, our findings support an evolutionary model in which the camel GS shares a common origin with the true stomach but has also acquired distinct specializations. The conserved cellular and molecular profiles enable the GS to perform core true stomach‐like functions during development. Concurrently, the unique epithelial subtypes in the GS and C3, characterized by high expression of pro‐proliferative and migratory genes, represent a key lineage‐specific innovation. We therefore propose that the GS evolves from a common ancestral gastric structure and subsequently undergoes specific specialization in camels, a process likely to be driven by these novel cell types to support its unique organogenesis and functional maintenance.

### 2.4 The Conservation and Divergence of C1

**FIGURE 4:**
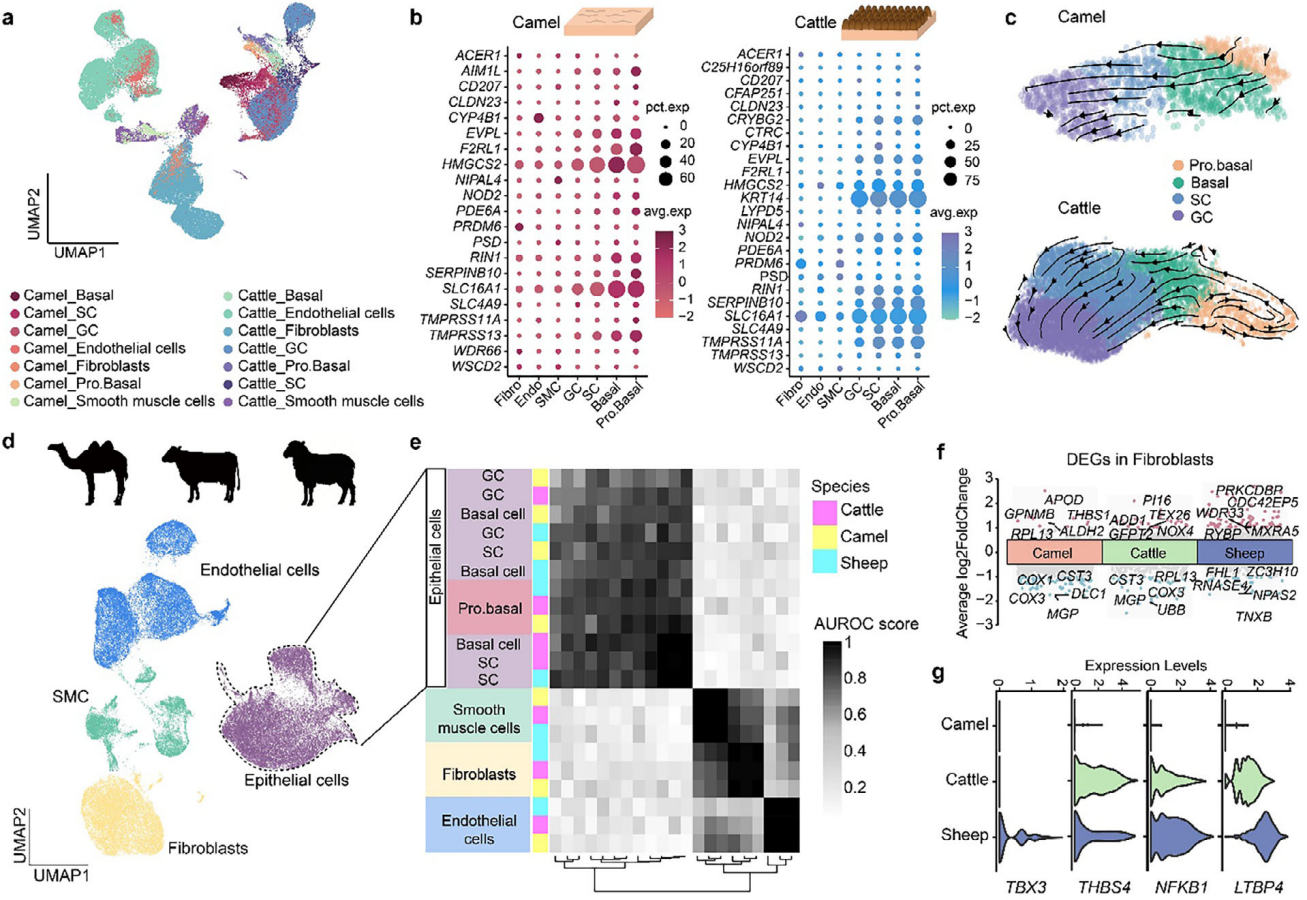
The cross‐species comparison of structural cell types in the C1 of camels, cattle, and sheep. a, A UMAP plot after integration of structural cells from the camel C1 and bovine rumen. Dot colors indicate different cell types. b, The dot plot showing the expression levels of 25 rumen core and positive selection genes in the structural cells from camel C1 (left) and bovine (right) rumen. c, The RNA velocity analysis of epithelial cells from the C1 of camels and cattle. d. A UMAP plot after integration of structural cells from the camel C1 and bovine and ovine rumen. Dot colors indicate different cell classes. e, Correlation of orthologous gene expression in structural cells among the C1 from camels and cattle. AUROC scores were used to measure the similarity of cell types: black, high correlation; grey and white, low correlation. f, The paired comparison of fibroblasts from the C1 of the three species, highlighting the top five upregulated and downregulated genes in each species. g, The expression levels of *TBX3*, *THBS4*, *NFKB*, and *LTBP4* in fibroblasts from the C1 of three species.

The C1 is the largest chamber in the multi‐chambered stomach system of both camels and ruminants, playing crucial roles in the primary processing and storage of food [32]. The results of hematoxylin and eosin (H&E) staining supported the classification of the epithelium in C1 of camels and cattle as keratinized stratified squamous epithelia (Figure S5a,b), despite significant differences in morphology and the structure of C1 across two species. The C1 region in camels included two adjunct GS areas, and the epithelial surface of the camel C1 appeared smooth and lacked papillary structures, distinct from the bovine rumen. In order to analyze the differences in gene expression profiles of structural cells between species and to evaluate the potential impact of interspecific cellular heterogeneity on the morphological differences in the epithelium of C1, we integrated single‐cell transcriptomic data of structural cells from bovine rumen and camel C1. The results showed a clustering pattern based on cell types (fibroblasts, SMCs, endothelial cells, and proliferating basal cells), indicating high conservation of these cell types between two species. Although the remaining epithelial cells also co‐clustered and exhibited highly similar gene expression profiles, there were no clear boundaries among the basal cells, spinous cells (SCs), and granular cells (GCs) (Figure 4a). Next, we delved into the molecular characteristics of structural cells in the bovine rumen and camel C1 from multiple viewpoints. As positive selection is one of the main drivers for evolution, explorations of the expression levels of positively selected genes in different cells could help identify the cell types on which natural selection acts preferentially. To this end, we examined the expression levels of 25 rumen core genes in the structural cells of C1. These genes have been reported to be positively selected in the common ancestor of ruminants [1]. We found that they were mainly upregulated in the epithelial cells (basal cells, SCs, and GCs) from camel C1 and bovine rumen, in particular *SLC16A1* and *HMGCS2*, which are associated with volatile fatty acid transport and ketone body synthesis. Other positively selected genes, such as *TMPRSS13*, *RIN1*, *F2RL1*, and *EVPL*, also showed similar upregulation in epithelial cell types compared to other structural cells (Figure 4b). The cellular biased expression pattern of positively selected genes indicates that natural selection plays an important role in molding the characteristics of the C1 epithelial cells, such as the high level of volatile acid absorption, in camels and cattle.

Keratinized stratified squamous epithelial cells are a category of constantly renewing cells [33]. To investigate interspecies differences in keratinocyte differentiation underlying epithelial renewal in the C1 from camels and cattle, we inferred the differentiation trajectory of the C1 epithelial cells in the two species by performing an RNA velocity analysis [34]. The results showed that all four epithelial cell types in the two species exhibited a clear trajectory, starting from proliferating basal cells and ending with GCs, which was consistent with the renewal of keratinocytes (Figure 4c). In addition, to examine whether this epithelial differentiation trajectory is conserved in other ruminant species, we incorporated published single‐cell transcriptomic data from the sheep rumen [26]. We first integrated structural cells from the C1 across camels, cattle, and sheep, and found that four major structural cell classes, i.e., fibroblasts, SMCs, endothelial cells, and epithelial cells, clustered by cell types, reflecting similarity in the gene expression profiles of structural cells across the three species (Figures 4d,e; Figure S5c). Consistently, epithelial cells from the sheep rumen displayed a differentiation trajectory characteristic of keratinocyte renewal (Figure S5d), indicating that the epithelial differentiation process in the C1 is highly conserved among these species. Then, we examined the expression trends of genes known to be related to keratinocyte differentiation and that represent unique functions of the rumen epithelium along the differentiation trajectory of the C1 epithelial cells in camels, cattle, and sheep (Figure S6a–c). The results showed that genes such as *STAT3* and *ZNF750*, which contribute to the differentiation of keratinocytes [35, 36], did not exhibit clear patterns of variation during the differentiation of epithelial cells in C1 across the three species. These findings indicate that while the direction of epithelial cell differentiation in C1 is similar across species, the driving molecular factors may differ among species. Notably, camel epithelial cells lacked the expression of *SLC14A1*, a gene responsible for urea reabsorption, while it was upregulated in basal cells and SCs from cattle and sheep. This expression pattern aligns with previous studies on *SLC14A1* [37], suggesting the accuracy of epithelial cell clustering in the C1 and the weakened urea reabsorption in the camel C1.

As fibroblasts have been reported to be direct regulators of keratinocyte differentiation [38], we then extracted and compared the fibroblast data from the C1 of camels, cattle, and sheep (Figure 4f). Across the three species, we identified 171 DEGs, including *TBX3* that has previously been reported to be involved in the early papillogenesis and papillae keratinization in ovine rumen [26]. The specific expression of *TBX3* in sheep suggests that the mechanism by which *TBX3* regulates rumen papillogenesis might be unique to sheep. Additionally, in the camel C1 epithelial cells, we found downregulation of genes such as *THBS4*, *NFKB1*, and *LTBP4*. Because upregulation of these genes in fibroblasts has been demonstrated to promote the proliferation and fibrosis of stratified squamous epithelia [39, 40, 41, 42], the reduced expression of these genes in the fibroblasts from the camel C1 might explain the absence of papillary structures in its epithelium (Figure 4g). Overall, these findings suggest that the cell types of the C1 epithelium are conserved, and that the fibroblasts are the primary cell type contributing to the formation of papillary structures in the C1 epithelium across the three species.

### 2.5 The Preference for Nutrient Absorption and Metabolism in Camels and Cattle

**FIGURE 5:**
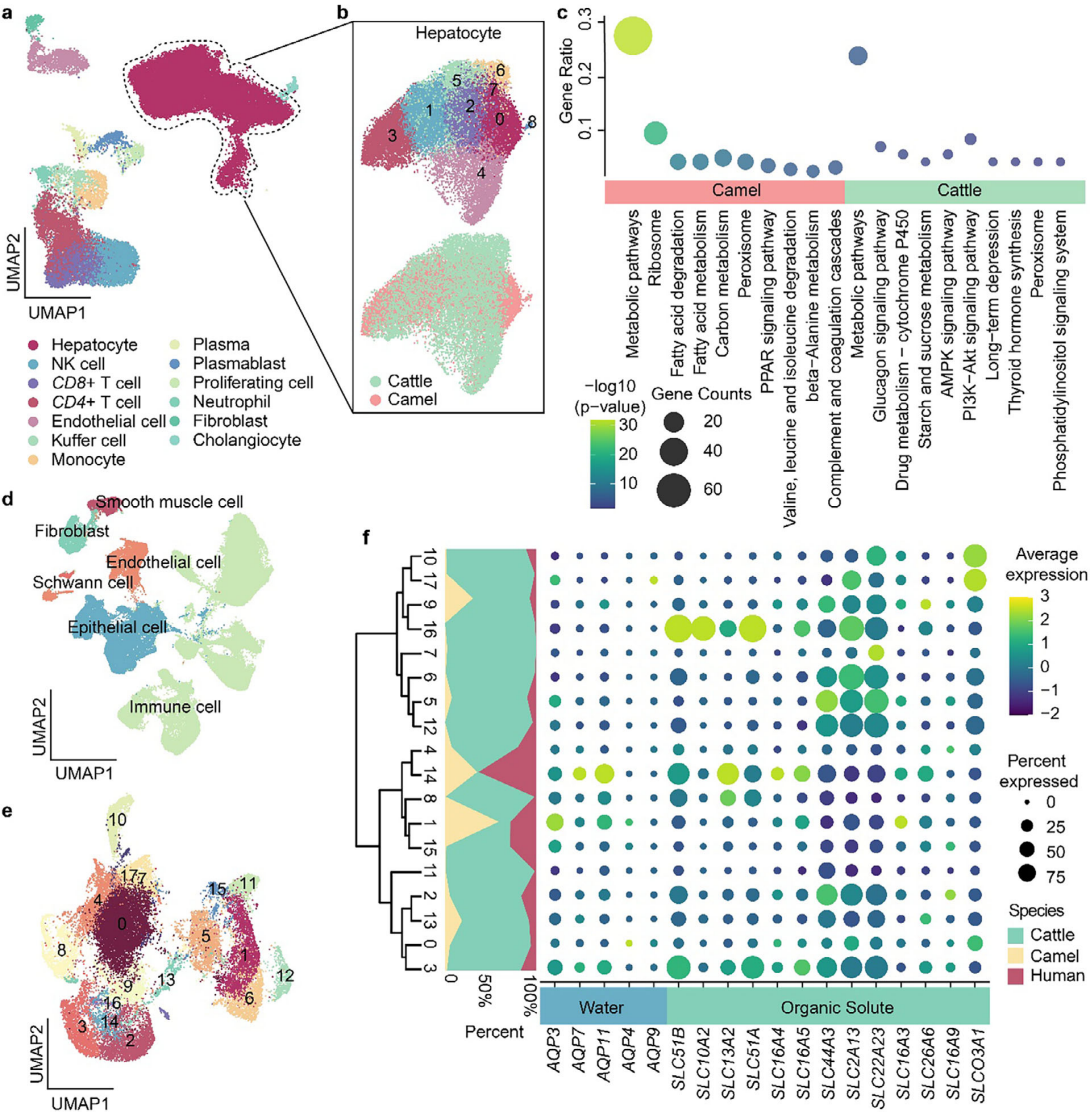
The cross‐species comparison of small intestines and livers across cattle, camels, and humans. a, Integration of scRNA‐seq data of livers from cattle and camels, and cell types are labelled with different colors. b, UMAP plots of hepatocytes from cattle and camels. c, The KEGG pathway enrichment analysis of species‐biased genes. d, Integration of scRNA‐seq data of small intestines from cattle, camels, and humans, and cell classes are labeled with different colors. e, A UMAP plot after integration of epithelial cells from small intestines in three species. Dot colors represent the different integrated clusters of epithelial cells. f, The branches on left represent the hierarchical clustering generated by the gene expression profiles of the integrated clusters. The graph on the middle represents the species mixture, and the right dot plot shows the expression level of genes related to water absorption and organic salt transport.

To assess the impact of forestomach specialization on the utilization of plant‐based diets, we extracted single‐cell transcriptomic data from the small intestines and livers of camels and cattle for cross‐species comparisons. We initially identified 13 major cell types in the livers of both species, among which hepatocytes represented the most abundant population (Figure 5a). Since hepatocytes constitute the principal site responsible for diverse nutrient metabolic processes in the liver [43], we next re‐clustered hepatocytes from both species at higher resolution and identified nine major hepatocyte clusters. Intriguingly, hepatocytes from camels and cattle did not segregate into species‐specific clusters (Figure 5b). To investigate functional specialization of hepatocytes between the two species, we further compared the expression patterns of all one‐to‐one orthologous genes in camel and bovine hepatocytes. We identified 286 camel‐biased genes and 72 cattle‐biased genes in hepatocytes (log_2_FC > 1, adjusted *p* < 0.05) (Table S9). The KEGG pathway enrichment analysis revealed that the camel‐biased genes were enriched with the fatty acid degradation/metabolism pathway, including *ACSL4*, *ACSL5*, and *ACOX1*, which are known to play key roles in the conversion of long‐chain fatty acids [44, 45]. These genes were also involved in the PPAR signaling pathway, a central regulator of lipid synthesis and metabolism [46], as well as in pathways related to amino acid metabolism. In contrast, cattle‐biased genes were significantly enriched with pathways such as drug metabolism, and starch and sucrose metabolism (Figure 5c). These expression biases suggest that camel hepatocytes preferentially support efficient lipid conversion and storage, whereas bovine hepatocytes are more involved in response to dietary fluctuations and processing exogenous compounds derived from feeds.

As the primary site for nutrient absorption and transport, the small intestine plays a critical role in determining dietary utilization efficiency across species [18, 47]. The human data served as an outgroup for identification of the shared characteristics of nutrient absorption in camels and cattle. The total 169,609 cells in the small intestines of three species were integrated by 13,695 one‐to‐one orthologs, resulting in 22 cell types (Figure 5d; Figure S7a and Table S10). The epithelial cells in small intestines were the major cell population responsible for nutrient absorption, which were further classified into five cell types shared by three species, namely enterocytes (*ALPI^+^*, *SLC26A3^+^*), goblet cells (*ZG16^+^*, *CLCA1^+^*), enteroendocrine cells (*CHGA^+^*, *CHGB^+^*, *CPE^+^*), transient‐amplifying (TA) cells (*MKI67^+^*, *TOP2A^+^*), and stem cells (*LGR5^+^*, *RGMB^+^*). These epithelial cells were first integrated and regrouped into 18 cell clusters for cross‐species comparisons, with over 200 cells in each cluster (Figure 5e). We then investigated expression profiles of genes encoding transporters for water, organic salt, inorganic salt, and sugar in 18 cell clusters. All analyzed transporter genes were verified as one‐to‐one orthologs across the compared species. This analysis revealed that the enterocytes from camels and humans were enriched in clusters 1 and 14, with highly expressed genes (*AQP3*, *AQP11*, and *AQP4*) involved in water absorption. There were no significant differences in the expression levels of transporters for inorganic salt and sugar among three species (Figure S7b), but for bovine enterocyte‐enriched clusters 10, 16, and 3, genes related to organic salt transport (*SLC51B*, *SLC10A2*, *SLC51A*, and *SLCO3A1*) were highly expressed. Some other genes involved in organic salt transport (e.g., *SLC44A3*, *SLC2A13*, and *SLC22A23*) were extensively detected in all cell clusters, while the elevated average expression levels of these genes were discerned in clusters 2, 5, 6, 12, and 16 that were enriched with bovine enterocytes and goblet cells (Figure 5f). Arguably, upregulation of genes related to organic salt transport in bovine enterocytes corresponds to the large number of volatile fatty acids produced by rumen microorganisms.

Together, these findings indicate the differential preferences for nutrient absorption and metabolism between camels and cattle, highlighting the advantage of camel livers in long‐chain fatty acid metabolism, which shields them from excessive fat deposition.

### 2.6 Vascular Smooth Muscle Cells (VSMCs) Showed Uncharacterized Heterogeneity in Camel Kidneys

**FIGURE 6:**
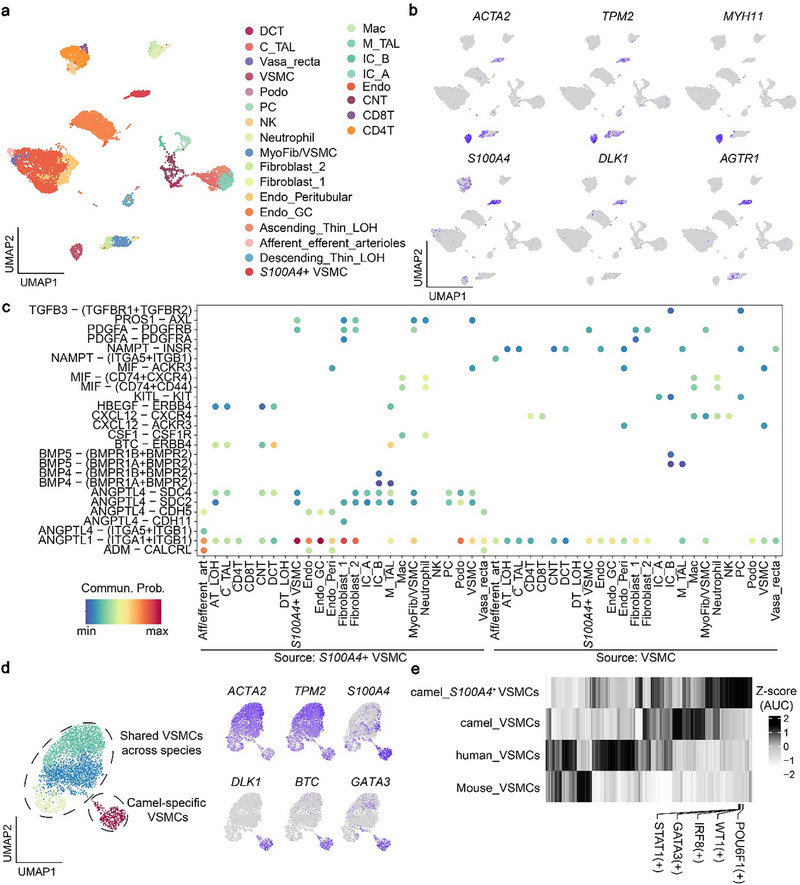
The markers and functional characteristics of *S100A4* ^+^ VSMCs in the camel kidney. a, The unsupervised clustering and cell type identification of 11,704 cells in the camel kidney. b, UMAP plots showing the expression of markers for contractile (*ACTA2*, *TPM2*, and *MYH11*) and *S100A4* ^+^ VSMCs (*S100A4*, *AGTR1*, and *DLK1*) in the camel kidney. c, Cell‐cell communication and signaling pathways between the two VSMC subtypes and other cell types. d, Integration of scRNA‐seq data of renal VSMCs from camels, humans, and mice. The UMAP plot on the left shows the cross‐species integration of VSMCs after unsupervised clustering, while the UMAP plots on the right are colored by the expression of markers for contractile and camel‐specific *S100A4* ^+^ VSMCs. e, Regulon activity scores of *S100A4* ^+^ VSMCs from the camel kidney and of contractile VSMCs from the kidneys of camels, humans, and mice. The top five regulons with the highest RSS values in *S100A4* ^+^ VSMCs are indicated. DCT: distal convoluted tubule; C_TAL: cortical thick ascending limb of henle's loop; Podo: podocyte; PC: principal cell; VSMC: vascular smooth muscle cell; Endo_GC: glomerular capillary endothelial cell; Mac: macrophage; IC_A/B: type a/b intercalated cell; Endo: endothelial cell; CNT: connecting tubule.

The kidney is the main organ that maintains fluid balance and that plays an important role in the adaptation of camels to high‐salt diets and arid environments [3]. To explore the main cell types and their molecular characteristics associated with the unique ability of camel kidneys, we focused on single‐cell transcriptomic data of camel kidneys, including 25 cell types (e.g., principal cells (PC), connecting tubule (CNT) intercalated cells, and afferent and efferent glomerular arteriolar endothelial cells) (Figure 6a). Of the stromal cell types, a cluster of cells expressing the markers of contractile VSMCs (*MYH11*, *ACTA2*, and *TPM2*) was identified. Intriguingly, we identified a VSMC cluster with high expression of *S100A4*, *AGTR1*, *ACTA2*, *TPM2*, and *DLK1*, hence referred to as *S100A4* ^+^ VSMCs. *AGTR1*, which encodes the angiotensin II type 1 receptor [48], was prominently expressed in these cells, highlighting an angiotensin II‐associated molecular signature (Figure 6b). Besides, *S100A4* ^+^ VSMCs displayed low expression of genes related to vasoconstriction (e.g., *MYH11*) but high expression of the key gene related to the SMC phenotypic transition, i.e., *S100A4*, distinct from the contractile VSMCs. To further characterize the functional properties of camel kidney VSMCs, we conducted an intercellular communication analysis between the two subtypes and other cell types. The results showed that the *S100A4* ^+^ but not contractile VSMCs specifically sent the signaling of *DLK1*‐*NOTCH3* and five *ANGPTL4*‐mediated signaling, which might be due to the high expression of *DLK1* and *ANGPTL4* in *S100A4* ^+^ VSMCs. It has been reported that *DLK1* mediates the phenotypic transition among various cell types (e.g., epithelial‐to‐mesenchymal transition, EMT) [49], while *NOTCH3* is mainly expressed in VSMCs and pericytes, especially in arterioles, responsible for maintenance of the structure and functions of VSMCs [50], and that *ANGPTL4* is involved in stabilization of atherosclerotic plaques and in modulation of the phenotypic transition of VSMCs [51]. Of the *S100A4* ^+^ VSMC‐specific ligand‐receptor pairs, *DLK1*‐*NOTCH3* and *JAG1*‐*NOTCH3* were found to drive the *S100A4* ^+^ VSMC‐to‐contractile VSMC signaling, and *ANGPTL4*‐*CDH5* ligand‐receptor pairs drove the *S100A4* ^+^ VSMC‐to‐endothelial cell signaling (Figure 6c; Figure S8). Hence, we identified a VSMC subtype (*S100A4* ^+^ VSMCs) with high expression of the angiotensin receptor genes but without the contractile ability, with the conjecture that the *DLK1*‐*NOTCH3* ligand‐receptor pair mediates the phenotypic transition of contractile to *S100A4* ^+^ VSMCs in the camel kidney.

To delve into the unique properties of *S100A4* ^+^ VSMCs in blood pressure regulation, we then inspected similar VSMCs that underwent the phenotypic transition in single‐cell transcriptomic atlases of kidneys from other mammalian species. First, we collected single‐cell transcriptomic data from human (n = 109,741) [52], murine (n = 28,214) [53], porcine (n = 6,938) [54], and bovine (n = 28,585) kidneys to construct a merged single‐cell transcriptomic atlas of mammalian kidneys, and examined the expression levels of markers for *S100A4* ^+^ VSMCs, including *DLK1*, *S100A4*, *AGTR1*, and *ACTA2*, to identify other similar VSMCs in the mammalian renal single‐cell transcriptomic atlases, if any. Surprisingly, no VSMCs similar to camel *S100A4* ^+^ VSMCs were found in the kidneys from other four mammalian species (Figure S9a–d). Subsequently, the 14,070 one‐to‐one orthologous genes among humans, mice, and camels were used to integrate all VSMCs from kidneys of three species (Table S11). After unsupervised clustering, we discerned that a co‐clustering of contractile VSMCs was shared in kidneys of the three species, along with the independent cluster of *S100A4* ^+^ VSMCs. Examination of genes expressed in *S100A4* ^+^ VSMCs also corroborated the uniqueness of camel renal *S100A4* ^+^ VSMCs and the utility of *DLK1* and *BTC* as specific markers for these cells (Figure 6d). Notably, while *AGTR1*, a key marker for this cell population, existed in a one‐to‐one orthology relationship among humans, cattle, and camels, its relationship with the mouse genome was one‐to‐many (due to lineage‐specific duplications giving rise to *Agtr1a* and *Agtr1b*). Therefore, *AGTR1* is not suitable for identifying a directly comparable cell type in mice. Nonetheless, the combined species‐specific expression pattern of other markers (*DLK1*, *BTC*, etc.) still defines this cell population at the transcription level. By analyzing the differences in regulons between *S100A4* ^+^ and contractile VSMCs from the kidneys of three species, we identified regulons with specifically high activity in *S100A4* ^+^ VSMCs, including *POU6F1*, *WT1*, *IRF8*, *GATA3*, and *SATA1* that ranked the top five for RSS (Figure 6e). These TFs have previously been reported to regulate the cell reprogramming and vascular remodeling [55, 56, 57, 58, 59], and we speculate that they play potential roles in the phenotypic transition of VSMCs in the camel kidney.

### 2.7 The Spatial Transcriptomic Analysis Disclosed the Diversity and Spatial Distribution of VSMCs in the Camel Kidney

**FIGURE 7:**
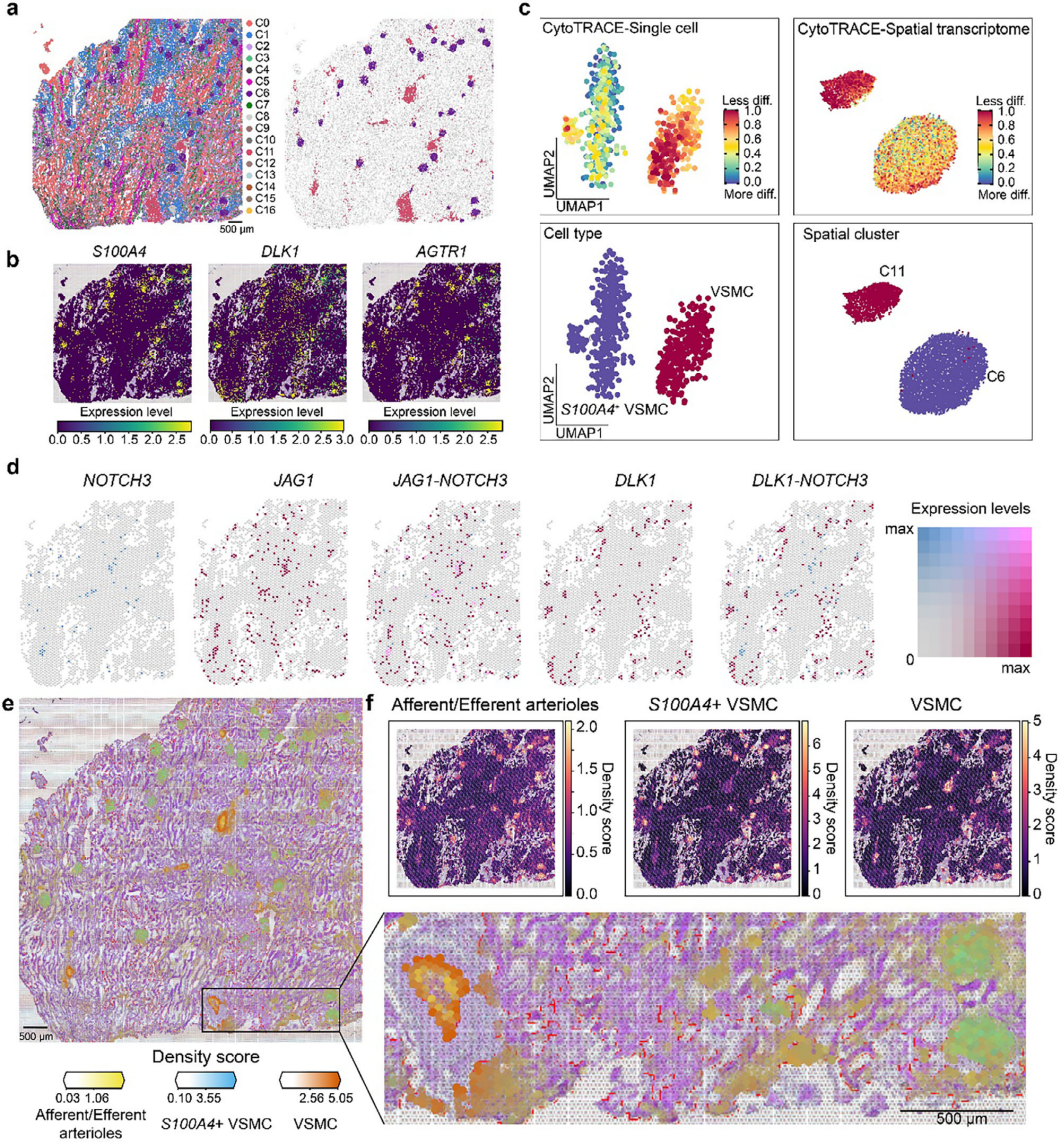
The spatial transcriptomic analysis of the camel kidney. a, Unsupervised clustering of spatial transcriptomic data from the camel kidney, with different colors representing different clusters (n = 17). Each irregular shape represents a spot after cell segmentation. The highlighted areas on the right indicate the locations of *S100A4* ^+^ (C6) and contractile VSMCs (C11). b, Spatially resolved expression of *S100A4* ^+^ VSMC markers (*AGTR1*, *DLK1*, and *S100A4*) in the camel kidney. c, The CytoTRACE analysis of contractile and *S100A4* ^+^ VSMCs in the scRNA‐seq data from the camel kidney (left) and the C6 and C11 in the spatial transcriptomic data (right). The dots in the upper UMAP show the differentiation score with different colors, while the lower UMAP depicts two different types of VSMCs. d, The co‐expression pattern of *DLK*‐*NOTCH3* and *JAG1*‐*NOTCH3* receptor‐ligand pairs in spatial locations. e, Cell2location mapping of camel renal cortex sections reveals the spatial matching of annotated cell types to their expected anatomical structures. Spatial cell density maps of a H&E‐stained kidney cortical section. The accompanying density maps of an H&E‐stained section display the distributions of afferent/efferent arterioles, *S100A4* ^+^ VSMCs, and VSMCs, distinguished by color. Scale bar, 500 µm. f, Hematoxylin‐eosin (H&E) staining images are overlaid with cell abundance density scores generated by cell2location, showing the distribution patterns of afferent and efferent arterioles, *S100A4* ^+^ VSMCs, and VSMCs cell types.

Further, to explore the spatial distribution of *S100A4* ^+^ VSMCs in the camel kidney, we collected a healthy camel renal tissue and performed a spatial transcriptomic analysis. Through the cellular segmentation algorithm [60], a total of 104,965 spots were obtained, with each spot containing spatial information and the corresponding gene expression profiles. After unsupervised clustering, these spots were assigned into 17 clusters (C0‐C16, Figure S10a). Of these, the most distinctive structure was identified as the glomerulus, which typically appears round or oblate in shape (Figure 7a). Besides, we identified C11, characterized by high expression levels of *ACTA2* and *MYH11*, as the contractile VSMC‐enriched cluster, whereas C6 with high expression levels of *S100A4* and *AGTR1* was identified as the *S100A4* ^+^ VSMC‐enriched cluster. The examination of the expression levels of markers for *S100A4* ^+^ VSMCs within each spot revealed that regions with high expression of *AGTR1*, *DLK1*, and *S100A4* overlapped with the spatial loci of C6 (Figure 7b and Figure S10b), consistent with the markers of two populations of VSMCs obtained from scRNA‐seq data. Additionally, by integrating spatial transcriptomic and scRNA‐seq data, we assessed the differentiation status of the camel contractile and *S100A4* ^+^ VSMCs, based on CytoTRACE scores [61]. Consistently, the *S100A4* ^+^ VSMCs were predicted to be more differentiated compared to the contractile VSMCs (Figure 7c). This is in line with the previous claim that VSMCs with distinct phenotypes originate from contractile and fibroblast‐like VSMCs [62]. To delve into the mechanisms for the phenotypic transition between contractile and *S100A4* ^+^ VSMCs in the camel kidney, we then explored the colocalization between receptor‐ligand pairs that had been identified by the scRNA‐seq analysis in the spatial transcriptome. The results showed that regions with high expression of *NOTCH3* (a key factor maintaining the normal vascular function and the VSMC phenotypic transition [50]) overlapped with the spatial loci of contractile VSMCs. What surrounded these areas were spots with high *DLK1* and *JAG1* expression, as well as those identified as *S100A4* ^+^ VSMCs (Figure 7d), suggesting direct interactions and a *DLK1*‐*NOTCH3*‐mediated phenotypic transition between these two VSMC subtypes. Hence, through the combination of spatial transcriptomic and scRNA‐seq data, we provided a proof for the presence of *S100A4* ^+^ VSMCs in the camel kidney.

The integrated analysis of scRNA‐seq and spatial transcriptomic data can significantly enhance the resolution. Hence, we employed cell2location [63] for spatial deconvolution to map cell types inferred from scRNA‐seq data onto the kidney spatial transcriptome. This analysis revealed that *S100A4* ^+^ VSMCs were consistently colocalized with specific endothelial populations associated with the afferent and efferent arteriolar regions (Figure 7e), in agreement with the prominent ANGPTL4‐mediated ligand–receptor interactions detected between *S100A4* ^+^ VSMCs and endothelial cells. Notably, both *S100A4* ^+^ VSMCs and canonical contractile VSMCs showed strong spatial association with glomerular capillary endothelial cells, consistent with their localization in vascular regions surrounding the glomerulus. Importantly, *S100A4* ^+^ VSMCs were barely detected in non‐glomerular vascular regions, indicating that their distribution is largely restricted to the glomerular microvascular niche rather than broadly across the renal vasculature (Figure 7f). This spatial confinement supports a localized regulatory role at the level of glomerular arterioles, rather than the general involvement in renal blood vessels. In parallel, the spatial transcriptomic analysis accurately recapitulated known renal anatomical organization: glomerular capillaries were specifically localized within the cortical labyrinth, whereas CNT intercalated cells and PC cells were confined to the medullary ray regions (Figure S10c). The faithful reconstruction of these well‐established anatomical features provides independent validation for the reliability and spatial accuracy of the deconvolution results. Collectively, these findings suggest that *S100A4* ^+^ VSMCs may modulate the local responsiveness of renal afferent and efferent arterioles to angiotensin II. Given the established role of glomerular arteriolar tone in controlling renal hemodynamics and, indirectly, systemic blood pressure, we interpret this mechanism as a plausible contributor to blood pressure regulation rather than a direct causal determinant.

## 3 Discussion

In this study, we presented the single‐cell transcriptomic atlases of 54 tissues from camels and cattle, delineating their major cell types. We further integrated previously published multi‐chamber stomach single‐cell transcriptomic datasets from cattle and sheep [11, 26], and kidney single‐cell transcriptomes from humans, cattle, pigs, and mice [11, 52, 53, 54]. This integrative analysis revealed camel‐specific cellular and molecular features in the digestive system, livers, and kidneys, providing new insights into the organization of the multi‐true stomach, hepatic lipid metabolism, and renal mechanisms potentially involved in blood pressure regulation.

This study also entailed extensive cross‐species comparisons at the single‐cell transcriptomic level. Similar to those in other cross‐species comparison studies [16], the comparisons here were based on 1:1 orthologous genes, which enables paired comparisons of genes across multiple species while ensuring high representativeness for gene expression patterns within each species. However, there are some weaknesses for this approach, e.g., some novel transcripts that play roles in cellular functional innovation and in organogenesis might be overlooked. In this regard, more accurate algorithms remain to be developed. Alternatively, precise genome resequencing could be used to obtain more sequence information and elucidate the functions of these novel transcripts in specific species. Other limitations, such as the absence of certain camel tissue samples and the relatively low biological replications, are also present in this study. These issues could be resolved in future studies by optimizing tissue lysis conditions and through the accumulation of more data. Nonetheless, these limitations have no evident influence on the overall quality of the constructed single‐cell transcriptomic atlas for the two species. Compared to those in the single‐cell transcriptomic atlases of other species (e.g., the porcine cell atlas: 20 tissues, 54 cell types [54]; the human cell atlas: 41 adult and 19 fetal tissues, 102 annotated cell types) [15], the number of cell types and the range of tissues covered in our study remain quite substantial. Despite this extensive coverage, a small subset of clusters remained with unassigned identities. These unassigned cells exhibited high expression of histone and ribosomal genes, suggesting a potentially actively proliferative state. However, given that their transcriptional profiles did not align closely with canonical markers for established cell types, we have designated them as unclassified in the current analysis to maintain data rigor. Overall, this multi‐tissue single‐cell transcriptomic atlas of the two species could provide a valuable resource for future in‐depth research and cross‐species comparisons.

Among other things, the camel digestive system is characterized by the unique and highly specialized multi‐chambered stomach system that consists of multiple true stomachs, being a special case in the mammalian digestive system. The GS areas and C3, capable of secreting digestive enzymes, prolong gastric digestion and enhance the efficiency of plant‐based food processing, reflecting the unique digestive adaptation in the camel multi‐chambered stomach [64, 65]. In contrast, in the *Artiodactyla*, the ruminants possess multiple stomach chambers, facilitating the digestion of plant‐based diets through microbial fermentation [66]. Our analysis showed that although the GS was anatomically distinct from the bovine abomasum, the two compartments were evolutionarily homologous and shared deeply conserved molecular programs for chemical digestion. Camels and cattle descended from a common artiodactyl ancestor, from which their gastric architectures diverged along distinct evolutionary trajectories. During this divergence, ruminants such as cattle underwent relative functional centralization, evolving a four‐chambered stomach in which chemical digestion became predominantly restricted to the abomasum. In contrast, the camel lineage followed a trajectory characterized by functional retention and expansion: the chemical digestive capacity was partially preserved in the C3 and further elaborated within the GS under a “3+1” stomach architecture. Consistent with this model, key gastric digestive genes, including *ATP4A* and *PGA5*, remained strict one‐to‐one orthologs between camels and cattle, indicating the reuse of an ancestral genetic toolkit within divergent structural contexts. Moreover, the identification of transcriptional regulators specifically active in the camel C3 and GS, such as *SOX8* and *UNCX*, suggests lineage‐specific regulatory remodeling that redistributed and amplified gastric digestive functions across homologous tissues. Together, these findings indicate that camel stomach evolution proceeds through functional expansion of homologous gastric compartments rather than the strict compartmentalization observed in ruminants, providing an alternative evolutionary strategy for efficient digestion of plant‐based diets. Together, these findings provide the first evidence for the origin of the camel GS, delineating a series of innovations in the digestive and metabolic organs of cattle and camels during evolution.

Camels can consume as many as eight‐fold more salt than sheep and cattle but without showing any signs or symptoms of hypertension [67], demonstrating the enormous potential of the camel kidney for maintenance of fluid homeostasis and regulation of blood pressure. Here, we identified a mechanism mediated by *S100A4* ^+^ VSMCs for regulation of the glomerular filtration rate and maintenance of vascular morphology, distinguishing them from other known VSMC subtypes [62]. The molecular signature of this population is highlighted by the high expression of *AGTR1* that serves as both a primary marker in our study and a critical effector within the renin‐angiotensin system (RAS) pathway. Cross‐species genomic comparisons revealed lineage‐specific evolutionary trajectories of the *AGTR1* gene family. In humans, cattle, and camels, *AGTR1* has retained its ancestral single‐copy state, resulting in a strict one‐to‐one orthologous relationship among these species. In contrast, a critical gene duplication event in the rodent ancestral lineage led to the expansion of the *AGTR1* family, giving rise to the paralogous genes *Agtr1a* (located on chromosome 13) and *Agtr1b* (located on chromosome 3). This fundamental difference in genomic architecture has endowed mouse models with a more complex and thus functionally redundant regulatory network of the renin‐angiotensin system.

The final integrative analysis of single‐cell and spatial transcriptomic data positioned *S100A4* ^+^ VSMCs at the afferent and efferent arterioles and revealed a distinctive molecular signature characterized by high *AGTR1* expression together with low expression of contractile genes. In light of their transcriptomic features and localization, we hypothesize that these cells may act as local modulators that buffer the vasoconstrictive effects of angiotensin II on neighboring contractile VSMCs, in a manner conceptually analogous to the pharmacological action of *AGTR1* receptor antagonists. Given the well‐established link between renal vascular dysfunction and hypertension [68], this putative mechanism suggests a potentially novel cellular pathway in blood pressure regulation that warrants future investigation.

## 4 Conclusion

To sum up, our study presented single‐cell transcriptomic atlases of multiple tissues from camels and cattle, along with spatial transcriptomic data of the camel kidney, providing valuable resources for investigation of species‐specific biological properties and their evolutionary history. Furthermore, by cross‐species comparisons of major digestive and metabolic organs such as the GS, kidneys, and livers, we provided evidence for the origin of camel GS from the C3, and disclosed that changes in gene expression of certain cell types prevent excessive fat deposition in the camel liver. Moreover, we identified a novel population of VSMCs in the camel kidney that may play a key role in renal adaptation to arid and high‐salt environments. Collectively, our findings illuminate the molecular and cellular basis of adaptive evolution in camels and offer new insights into the evolutionary diversification of mammalian organ systems.

## 5 Experimental Section

### 5.1 Ethics declarations

All experimental procedures in this study were strictly adhered to local legislations and approved by the Institutional Animal Care and Use Committee of Northwest A&F University (Approval No. XN2024‐0416). The sampling process was rigorously monitored by members of the Institutional Animal Care and Use Committee of Northwest A&F University, ensuring the minimal distress of animals during experimentation.

### 5.2 Collection of Camel and Bovine Tissues

We collected samples from three female (approximately 22‐month‐old) and two male Holstein dairy cattle (aged 40 days, and 5 months, respectively) sourced from a local slaughterhouse in Xi'an, China. In addition, three female (approximately 3‐year‐old) and three male Bactrian camels (aged approximately 10 days, 6 months, and 20 months, respectively) were obtained from a slaughterhouse located in Gansu, China. The Holstein dairy cattle aged 40 days and 5 months, as well as the camels aged 10 days and 6 months, were euthanized due to severe and incurable trauma. Then, bovine and camel tissues were isolated, quickly rinsed with the pre‐cooled solution of 1 × PBS (Ambion, #AM9624), and placed on an ice‐cold board for dissection. Each tissue (except peripheral blood) was cut into 5–10 pieces, with each piece weighing 50–200 mg. Next, the fresh samples prepared for scRNA‐seq (e.g., livers, kidneys, multi‐chambered stomachs, and testes) were transferred to MACS Tissue Storage Solution (Miltenyi Biotec Technology & Trading, #130‐100‐008) and stored at 4°C, and these samples were dissociated within 48 h. After tissue dissociation, cell suspension was filtered by cell strainers after debris removal and erythrocyte lysis. The dissociation of PBMCs was attained by using a bovine peripheral blood extraction kit (Beijing Solarbio Science & Technology, #P8640), following the manufacturer's instructions. The samples prepared for snRNA‐seq (e.g., hump fat, tongue, frontal lobe, and cerebellum) were subjected to the uniform procedure, and then placed into cryovials (Corning, #430659) and stored in liquid nitrogen until lysis for nucleus extraction. For the spatial transcriptomic analysis, kidney samples were obtained from an adult female camel. The tissues were initially sectioned into approximately 6 × 6 mm^2^, and the edges were neatly trimmed to control the thickness of tissue blocks (around 3 mm). Then, the tissue surface was washed with pre‐cooled 1 × PBS. Small fragments of tissues were snap‐frozen in liquid nitrogen and embedded in the optimum cutting temperature (OCT) compound (SAKURA, #4583), and then stored at ‐80°C for further use. Subsequently, a representative cryosection containing the renal cortex was selected for the spatial transcriptomic library preparation. The detailed sampling locations for all tissues involved in this study, as well as the tissue dissociation strategies applied to each, are listed in Table S1.

### 5.3 Single‐Nucleus/‐Cell Suspension Preparation

Single‐nucleus/‐cell isolation was performed as previously described [47, 54]. In brief, frozen tissues were homogenized in a homogenizer in 1 mL lysis buffer consisting of 250 mM sucrose (Ambion), 10 mg/mL bovine serum albumin (Ambion), 5 mM MgCl_2_ (Ambion), 0.12 U/µL RNasin Plus (Promega, #N2115), and 1 × Complete Protease Inhibitor Cocktail (Roche, #11697498001). The tissue underwent two additional rounds of homogenization and processing for uniformity, and the homogenized tissue was subjected to a 40 µm cell strainer. The filtered nuclei were pelleted at 500 × g for 5 min at 4°C, and the pellets were resuspended in 1 mL buffer containing 10 mg/mL BSA, 3 mM CaCl_2_, 10 mM Tris‐HCl, 320 mM sucrose, 0.1 mM EDTA, 2 mM magnesium acetate, 1 mM DTT, 1 × Complete Protease Inhibitor Cocktail, and 0.12 U/µL RNasin, followed by centrifugation at 500 × g for 5 min at 4°C to pellet the nuclei. The isolated nuclei were then used for library preparation.

For cell suspension preparation, the fresh samples were transferred into a 50 mL tube with 20 mL digestion medium containing 0.5 mg/mL collagenase type II (Gibco, #17101015), 1.25 mg/mL protease (Sigma, #P5147‐100MG), and 7.5 µg/mL DNase I (Sigma, #D4527‐10KU) in cold HBSS. The tissue digestion was conducted at 37°C for 15 min, with gentle shaking every 5 min. The reaction was stopped by adding 20 mL of cold MACS buffer containing 0.25% BSA (Sigma‐Aldrich, #10735096001) and 2 mM EDTA in PBS. Subsequently, the mixture was filtered using a 100 µm cell strainer (Sigma‐Aldrich, #CLS431752‐50EA), and the obtained cells were prepared for the library construction.

The DNBelab C Series Single‐Cell Library Prep Set (MGI, #1000021082) was employed, following previously established protocols [47]. Specifically, the single‐nucleus/‐cell suspension was first subjected to droplet generation, emulsion breakage, bead collection, reverse transcription, and cDNA amplification to generate barcoded libraries. After the quality control of obtained cDNAs, indexed libraries were constructed according to the manufacturer's protocol. Subsequently, sequencing was conducted on the DNBSEQ‐T1 platform.

### 5.4 Spatial Transcriptomic Sequencing

After optimization of permeabilization, the frozen tissue in a pre‐cooled cryostat was cut into the thickness of 10 µm and placed on chilled BMKMANU S1000 Tissue Optimization Slides and BMKMANU S1000 Gene Expression Slides that are developed by BMKGENE (https://www.bmkgene.com/), and these slides were stored at ‐80°C until use. Spatial transcriptomic slides were printed with 1–8 identical capture areas (6.8 × 6.8 mm^2^), each with 2,000,000 spots that contain barcoded primers (BMKMANU S1000). In addition, fixation, staining, and imaging were performed on the sectioned slide as previously described [69]. Specifically, the tissues were fixed with 3.7‐3.8% formaldehyde (Sigma‐Aldrich) in PBS (Medicago) for 30 min, and then washed with 1 × PBS (Medicago). For staining, sections were incubated with Mayer's hematoxylin (Dako, Agilent, Santa Clara, CA) for 4 min, with bluing buffer (Dako) for 30 s, and with Eosin (Sigma‐Aldrich, a 1: 5 dilution) in Tris‐base (0.45 M Tris, 0.5 M acetic acid, pH 6.0) for 30 s. After air‐drying, bright‐field (BF) images were taken at 20 × magnification using the Metafer Slide Scanning platform (MetaSystems). Next, the reverse transcription and spatial library preparation were performed according to the standard protocol. Sequencing was performed on the Illumina NovaSeq 6000 with the sequencing depth of at least 50,000 reads per spot (100 µm) and 150 bp (PE150) paired‐end reads.

### 5.5 Spatial Transcriptomic Data Processing

We conducted an upstream analysis using BSTMatrix (v2.3), aligning the data to the reference Ca_bactrianus_MBC_1.0 camel genome. The count matrices generated were utilized for the downstream analysis. The cell segmentation was conducted using the built‐in “cellpose” function within BSTMatrix, and the corresponding Seurat (v.4.3.0) [70] object was generated as output. Additionally, spatial transcriptomic images and Seurat objects at resolutions of levels 7 (resolution = 50 µm) and 13 (resolution = 100 µm) were created to detect gene expression and for the integrated analysis with single‐cell transcriptomic data. Subsequent analyses of Seurat objects included normalization, clustering, and identification of markers using Seurat (v.4.3.0).

### 5.6 Processing of scRNA‐seq and snRNA‐seq Data

The data from scRNA‐/snRNA‐seq were filtered, and the gene expression matrix was obtained using the DNBelab C Series scRNA analysis software (v1.0.1, MGI) (https://github.com/MGI‐tech‐bioinformatics/DNBelab_C_Series_scRNA‐analysis software). The reference genomes of cattle (ARS‐UCD1.2) and camels (Ca_bactrianus_MBC_1.0) used for sequence alignment were downloaded from the Ensembl database and NCBI, respectively. Cells were only retained if the number of detected genes was greater than 200 and less than 5,000 and the percentage of detected mitochondrial transcripts from MT genes was less than 10.

Then, we created a Seurat object using the Seurat from three tables including genes, barcodes, and the raw UMI count generated by the DNBelab C Series scRNA analysis software (v1.0.1). All Seurat‐based analyses were performed using Seurat v4.3.0, where the “NormalizeData, ScaleData and FindVariableFeatures” functions were used to standardize the raw count matrix and identify the top 2,000 most variable genes. The “RunPCA, RunUMAP, FindNeighbors, and FindClusters” functions were used to reduce dimensionality and for clustering of data. “FindAllMarkers and FindMarkers” functions were used to identify DEGs across or between clusters with the options “min.pct = 0.25, logfc.threshold = 0.25”. Multiple test correction for *p* value was performed using the False Discovery Rate (FDR) method, and *P*. adj. < 0.05 was considered statistically significant. Further, cell‐type identities were assigned using canonical cell‐type markers.

The intercellular communication analysis was conducted using the R package CellChat (v1.6.1) [71] with default parameters, based on annotated cell types using the camel kidney scRNA‐seq dataset. We employed the “netVisual_bubble” function to visualize multiple ligand‐receptor‐mediated cellular interactions. Visualization was mainly fulfilled through the R package ggplot2 (v3.5.1).

### 5.7 Cell Annotation and Classification

The definitions of cell classes and cell types were established through an iterative clustering strategy. First, all cells from a given tissue were subjected to an initial round of dimensionality reduction and clustering. Based on highly expressed genes within each cluster, cells were broadly classified into major cell classes, such as epithelial cells, endothelial cells, fibroblasts, SMCs, and immune cells. Subsequently, cells belonging to each cell class were extracted and re‐embedded for further dimensionality reduction and clustering. Markers highly expressed in each resulting cluster were then identified, allowing us to define higher‐resolution cell types, such as neck cells, basal cells, and T cells.

For cell subtypes defined by specific markers (e.g., *S100A4*), we first determined the corresponding cell types through within‐species clustering and annotation. We then performed cross‐species integration among the same cell types to identify genes that were specifically and consistently expressed within these populations. Based on the combined evidence from within‐species clustering and cross‐species integration, we defined these marker‐based cell subtypes. In cases where cross‐species integrated clustering did not yield distinct cell clusters supported by highly variable genes, we named these cell subtypes according to the integrated cluster identifiers.

### 5.8 Cross‐Species Comparisons of Single‐Cell Transcriptomic Data

We followed previously established approaches for ortholog identification in cross‐species single‐cell transcriptomic studies [72]. Orthologs for camel, cattle, mouse, and human genes were independently identified using EggNOG‐mapper (v2.1.4) [13] that assigns optimal predicted gene names based on protein sequence homology in the EggNOG database. The parameters were set as follows: taxonomic scope = Mammalia, orthology restriction = all, with all other parameters left at default. From the resulting “.emapper.orthologs” files, proteins annotated with the one‐to‐one label in the orth_type field were extracted. These candidates were further manually curated based on predicted gene names to generate the final gene sets used for cross‐species comparisons.

Using this strategy, we identified 15,324 one‐to‐one orthologs, 464 one‐to‐many, 477 many‐to‐one, and 145 many‐to‐many orthologous relationships between cattle and camels. For comparisons involving three or more species, we calculated orthology relationships for all pairwise species combinations and selected genes that satisfied the one‐to‐one criterion across all relevant pairs as the primary gene set for integration. Other orthologs were retained if they appeared in at least one species pair. Ultimately, this yielded 14,070 one‐to‐one orthologs, 398 one‐to‐many, 1,486 many‐to‐one, and 570 many‐to‐many relationships among camels, mice, and humans, and 13,695 one‐to‐one, 762 one‐to‐many, 652 many‐to‐one, and 197 many‐to‐many relationships among humans, cattle, and camels.

For cross‐species comparisons of scRNA‐seq data from tissues such as livers, multi‐chambered stomachs, small intestine, and kidneys, the first step involved gene name conversion based on one‐to‐one orthologs. Subsequently, integration and batch removal of scRNA‐seq data from different species were carried out using the “RunHarmony” function within the R package Harmony (v.0.1.1) [73]. SAMap (v.1.0.15) [17] was used to integrate and align single‐cell transcriptomic data from camels and cattle. The alignment scores generated by SAMap reflect the transcriptional similarity between cell lineages, with higher scores indicating greater similarity. Cross‐species DEGs were identified using the “FindMarker” function in Seurat, with KOBAS (v3.0) used to identify associated GO terms [74].

### 5.9 Identification of TF Modules

The SCENIC (v1.3.0) [19] was employed to assess the regulatory activities of each single cell. Following the exclusion of regulons active in less than 7% of cells in the dataset, tables containing RSS and regulon activity scores were obtained. These tables were utilized for subsequent analyses of regulon modules and correlations. The RSS matrix was further utilized to filter species‐specific TFs. After computation of Spearman correlation coefficients among regulon activity scores for different cell types, hierarchical clustering was performed using hclust.method = “ward.D2”, and the regulons were partitioned into different modules. For each regulon containing a set of genes, the Seurat (v.4.3.0) “AddModuleScore” function was used to calculate expression scores for gene sets at the single‐cell level.

### 5.10 Species‐Specific Pseudo‐Bulk Analysis

Pseudo‐bulk samples were generated using the Seurat Average Expression function with various groups of cells dependent on the pseudo‐bulk samples produced in the study. The method for calculating the “pairwise gene expression distance” across species referred to previously published studies [16]. In short, by using the tissue gene expression profile obtained from pseudo‐bulk, the Spearman correlation coefficient between two tissues was calculated. This similarity coefficient was then converted to an expression distance as 1 − ρ. A smaller distance indicates that the gene expression program of the tissue is more evolutionarily conserved, while a larger distance suggests that it may have undergone faster transcriptomic remodeling.

### 5.11 RNA Velocity Analysis of Epithelial Cells and Stemness Inference of VSMCs

The scanpy (v1.10.3) [75] and scVelo (v0.3.2) [34] were employed to infer the developmental trajectory of single cells, and the RNA velocities of cells were estimated by quantifying unspliced and spliced mRNAs, based on the BAM file and the annotated cell types. Additionally, the R package CytoTRACE (v0.3.3) [61], a tool for the unbiased prediction of differentiation in scRNA‐seq data, was used to infer the stemness of VSMCs.

### 5.12 Spatial Cell Type Deconvolution Analysis

Spatial cell type deconvolution was performed using the cell2location framework (v0.1.3) [63]. First, a reference scRNA‐seq signature matrix was built from the annotated camel kidney dataset comprising 25 distinct cell types. The scRNA‐seq data (count matrices, cell barcodes, and gene features) were loaded using Scanpy (v1.10.3) [75], and a regression model (cell2location.models.RegressionModel) was trained for 150 epochs on the reference data using cell type labels to infer cell type‐specific gene expression profiles. For spatial transcriptomic data, the raw counts and spatial coordinates were preprocessed and aligned with the same gene annotation. The trained reference model was then used to deconvolve the spatial transcriptomic spots, estimating the posterior distributions of cell type abundances at each location. Low‐confidence predictions were filtered out by retaining only cell types whose fifth percentile of the posterior distribution (Q05) exceeded 0 cells per spot and whose maximum Q05 across all spots was ≥ 2 cells, ensuring robust spatial mapping. The resulting cell type abundance estimates (means and 95% credible intervals) were visualized in spatial context using the cell2location plotting utilities, with spot size and transparency adjusted for clarity. All analyses were conducted in Python (v3.10.12).

## Author Contributions

Y.J. and T.S. conceived and designed the project. T.S., H.Shan, H.W., and X.G. performed single‐cell and spatial transcriptomic analyses. Z. N., F.W., G.T., J.R., Y.Zhou, W.H., A.Z., and X.C. helped with bioinformatic analyses. H.L., B.H., S.Z., Q.Z., W.Z., and M.J. contributed to cell type annotation. T.S., H.Shan, H.W., and X.G. provided critical intellectual input and data interpretation. H.Sun, D.S., X.W., L.F., Y.Zheng, and Y.J. provided useful feedback and discussions. Y.J. provided funding, discussion, and supervision. T.S., Y.Zheng, and Y.J. prepared the manuscript with input from all authors.

## Funding

This work was supported by the National Key R&D Program of China (2022YFF1000100), the Shaanxi Livestock and Poultry Breeding Generic Technology Research and Development Platform (2023GXJS‐02), the Key Science and Technology Special Project of Xinjiang Uygur Autonomous Region (2024A02004‐2), and the Shaanxi Laboratory Project for Arid Region Agriculture (2024ZY‐JCYJ‐02‐15).

## Conflicts of Interest

The authors declare no conflict of interest.

## Supporting information




**Supporting File**: advs74150‐sup‐0001‐SuppMat.docx.

**Supporting File**: advs74150‐sup‐0002‐Table S1–S11.xlsx.

## Data Availability

The data that support the findings of this study are openly available in [Sequence Read Archive] at [https://www.ncbi.nlm.nih.gov/bioproject/PRJNA1119173; https://www.ncbi.nlm.nih.gov/bioproject/PRJNA1112075], reference number [1112075].
