# Supplementary material for: Single‐Cell Transcriptomic Atlases of Camels and Cattle Unravel Molecular Evolution of Digestive and Metabolic Systems
Source: Adv Sci (Weinh). 2026 Feb 3;13(20):e19346. doi: 10.1002/advs.202519346 (PMC13067795; doi:10.1002/advs.202519346)
Supplement: Supplementary file 1 — Supporting File: advs74150‐sup‐0001‐SuppMat.docx. [file ADVS-13-e19346-s001.docx]

**Supplementary information**

**Single-Cell Transcriptomic Atlases of Camels and Cattle Unravel Molecular Evolution of Digestive and Metabolic Systems**


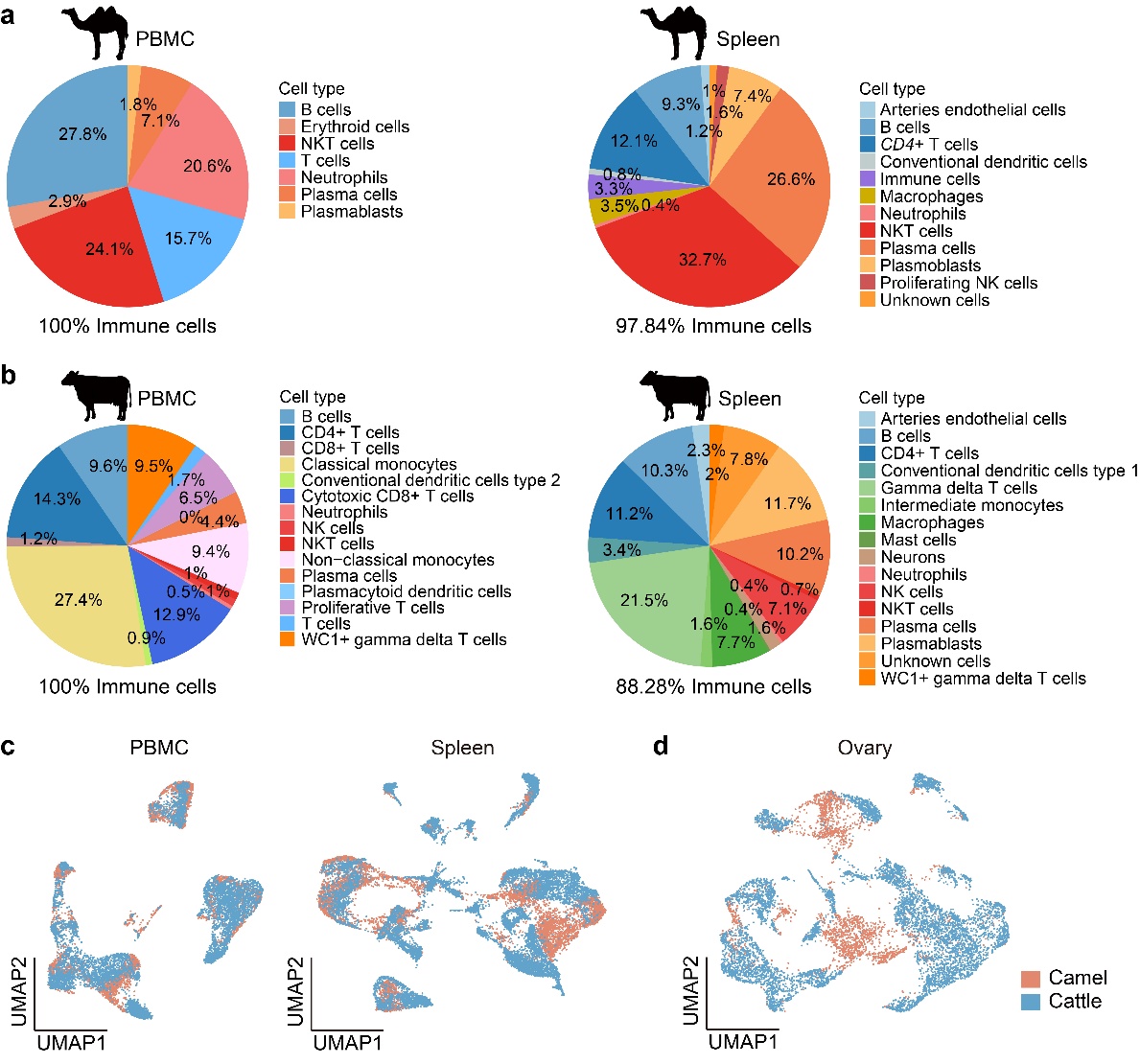


**Supplementary Fig. 1: The cross-species comparison of PBMCs, spleens, and ovaries from camels and cattle. a**, **b**, The cell types and their proportions in PBMCs and spleens from camels and cattle. **c**, **d**, The integration of cells from PBMCs, spleens, and ovaries in camels and cattle. Different colors of the dots represent the cells from camels (orange) and cattle (blue).


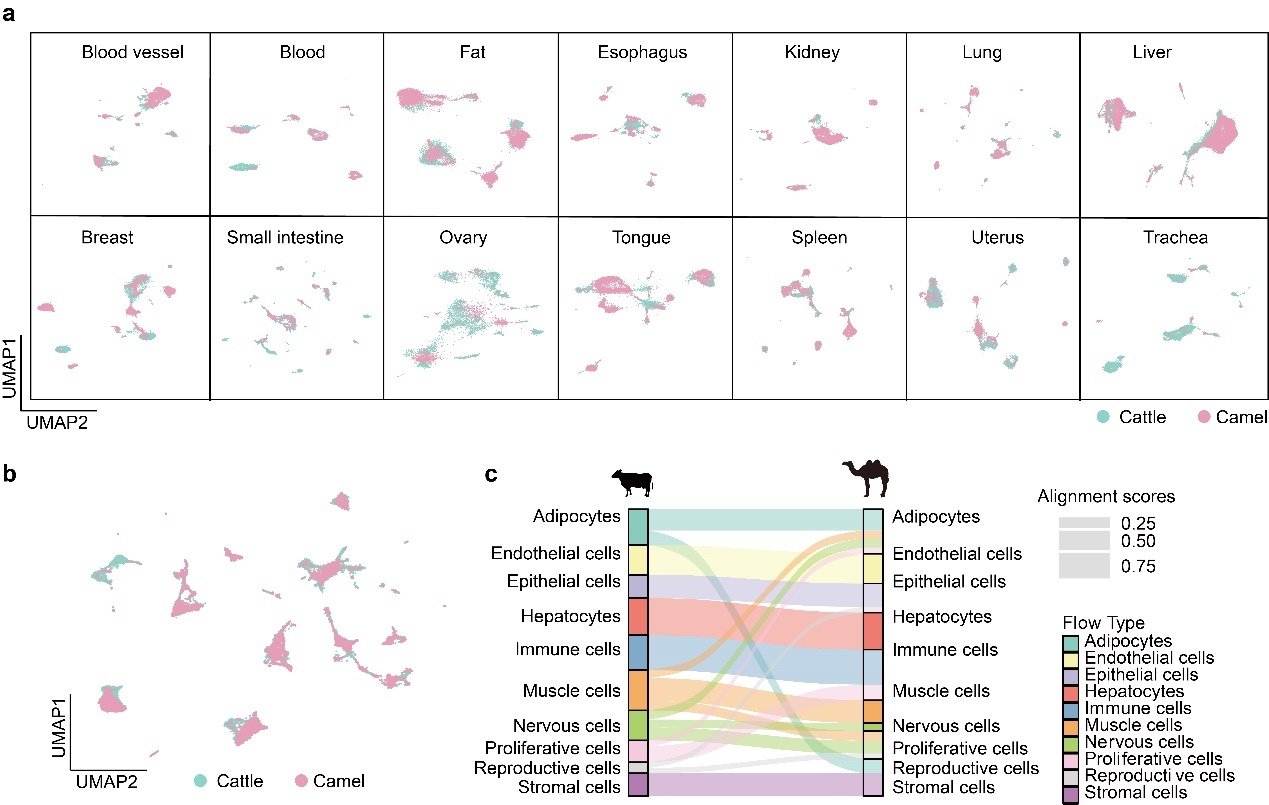


**Supplementary Fig. 2: The paired comparison of cell classes and tissues between camels and cattle. a**, Mapping of cells between the corresponding 14 tissues from camels and cattle by the SAMap pipeline. **b**, Integration of cell classes from camels and cattle, with each cell class downsampled to 500. **c**,The pairwise comparison of each cell class between camels and cattle, and the thickness of the flow represents the alignment score of the cell classes between the two species, where thicker lines indicate higher similarity between the two linked cells.


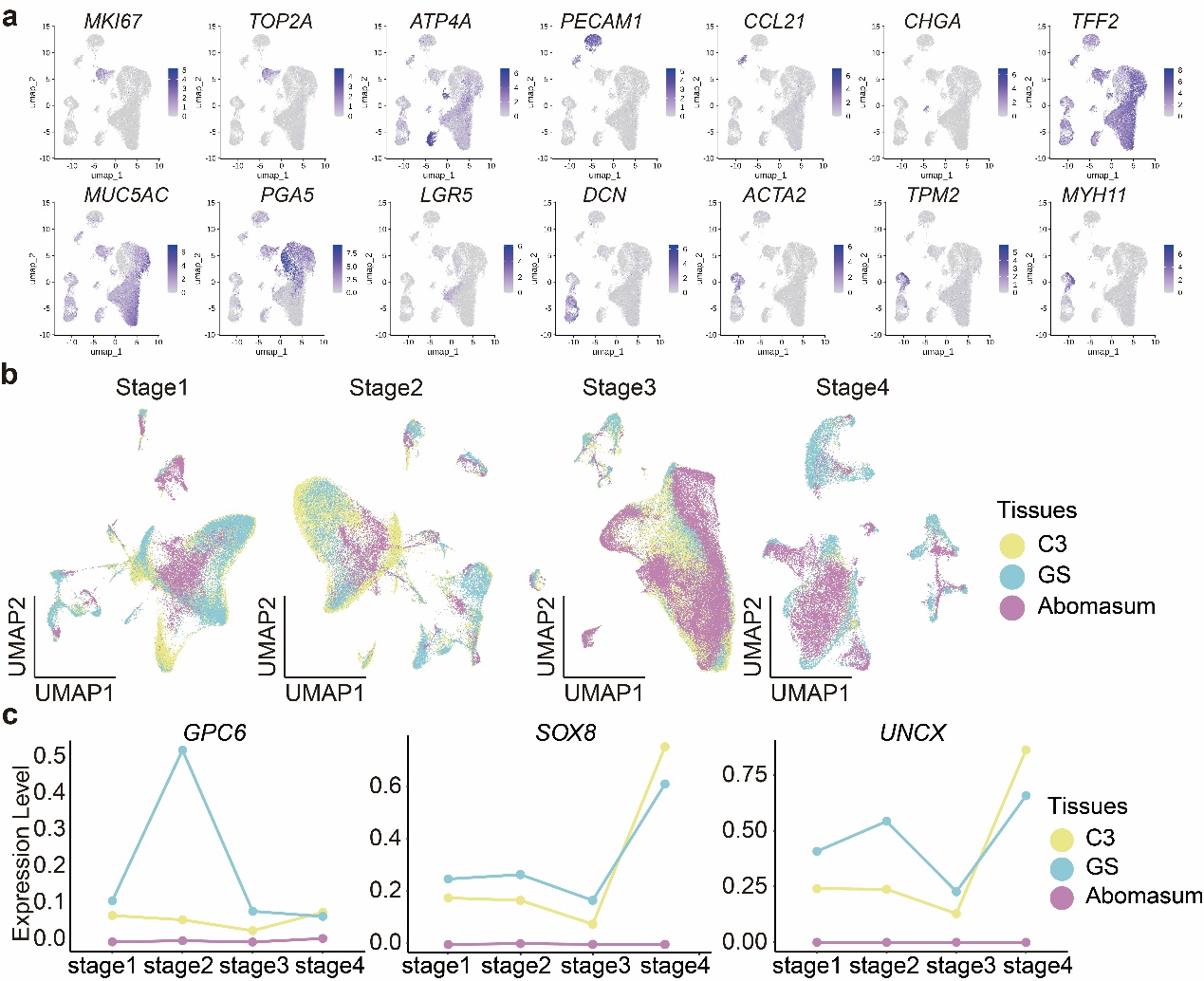


**Supplementary Fig. 3: Gene expression and integration of GS, C3, and abomasum at four developmental stages. a**, UMAP plots colored by the expression of markers used for cell type annotation. **b**, Independent integration of structural cells from the GS, C3, and abomasum at each developmental stage (Stages 1-4). **c**, Developmental stage-dependent expression of *GPC6*, *SOX8*, and *UNCX* in epithelial cells from the GS, C3, and abomasum.


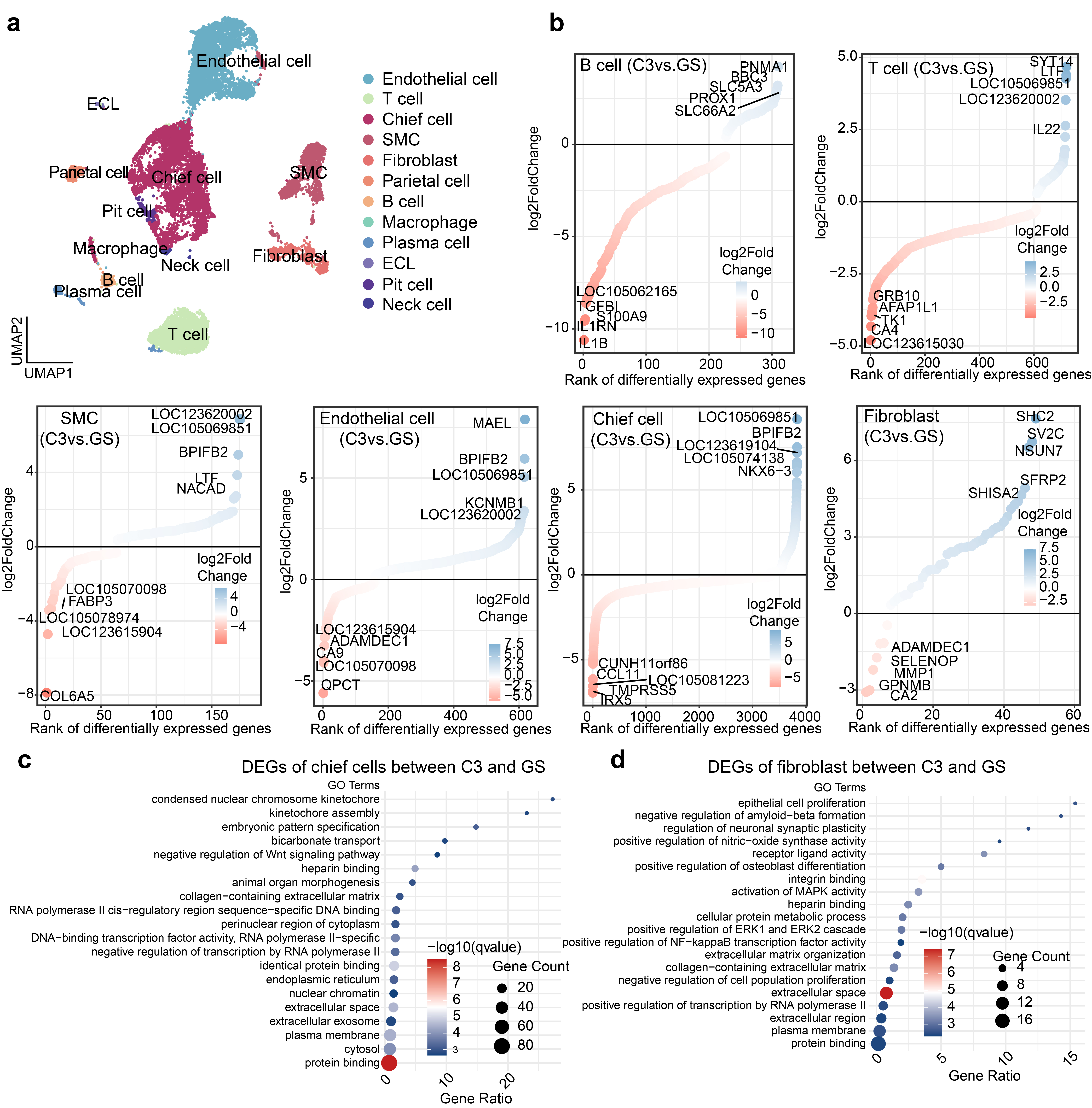


**Supplementary Fig. 4: The heterogeneity of structural cells in camel C3 and GS. a**, An UMAP plot of cell types from camel C3 and GS; **b**, DEGs in each cell type (with more than 200 cells) between C3 and GS; **c**, **d**, The GO enrichment analysis of DEGs in chief cells and fibroblasts between C3 and GS.


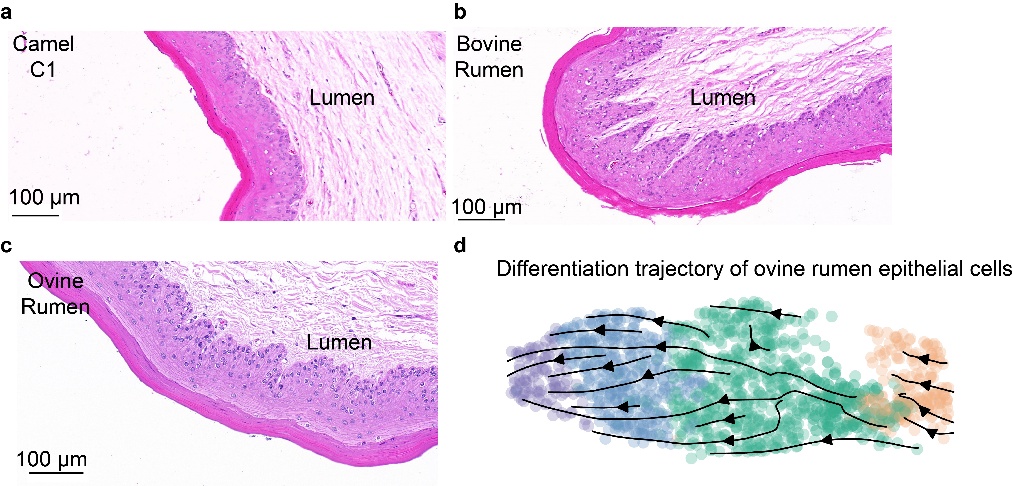


**Supplementary Fig. 5: The morphology of the epithelial tissues in camel C1 and bovine and ovine rumen. a**, **b**, Hematoxylin and eosin (H&E) staining of epithelial tissues from camel C1 (**a**) and bovine (**b**) and ovine (**c**) rumen. Bar = 100 µm. **d**, The RNA velocity analysis of epithelial cells from the ovine rumen.

**
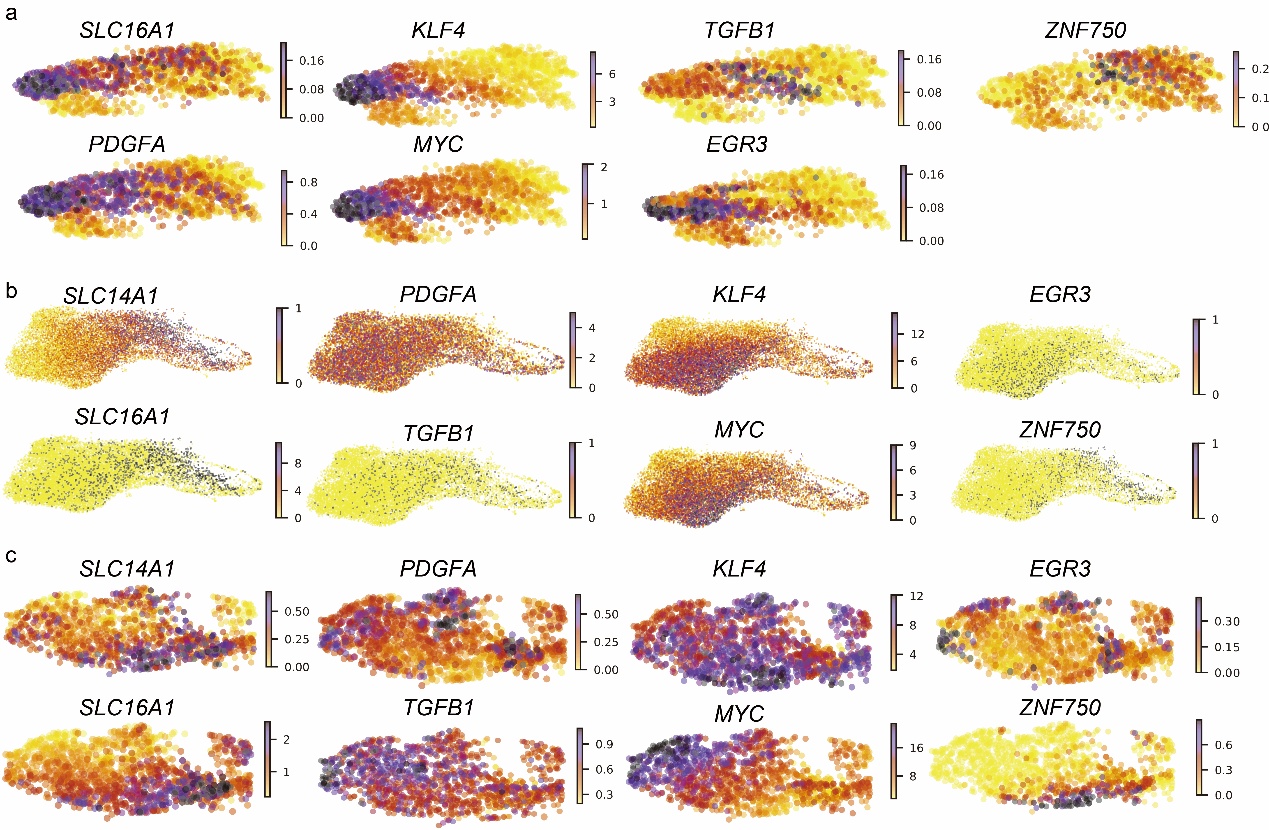
**

**Supplementary Fig. 6:** The expression levels of *MYC*, *KLF4*, *SATA3*, *ZNF750*, and *EGR3* along the differentiation trajectory of epithelial cells from camel C1 (**a**) and bovine (**b**) and ovine (**c**) rumen.


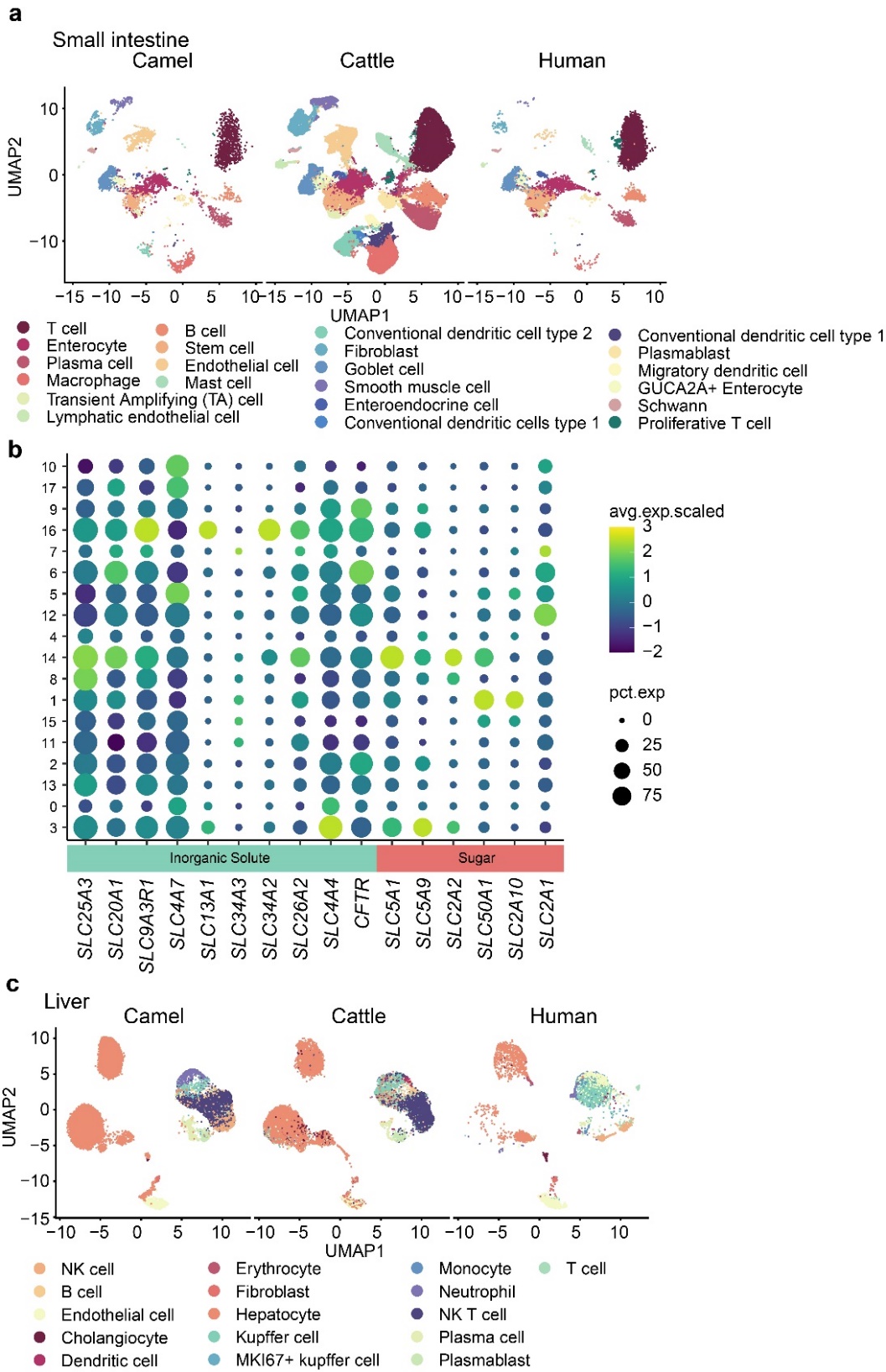


**Supplementary Fig. 7:** **The cell types in the small intestines of camels, cattle, and humans. a**, UMAP plots representing the integration of cells in small intestine from camels, cattle, and humans. **b**, The dot plot showing the expression level of genes related to inorganic salt and sugar transport in small intestine epithelial cells.


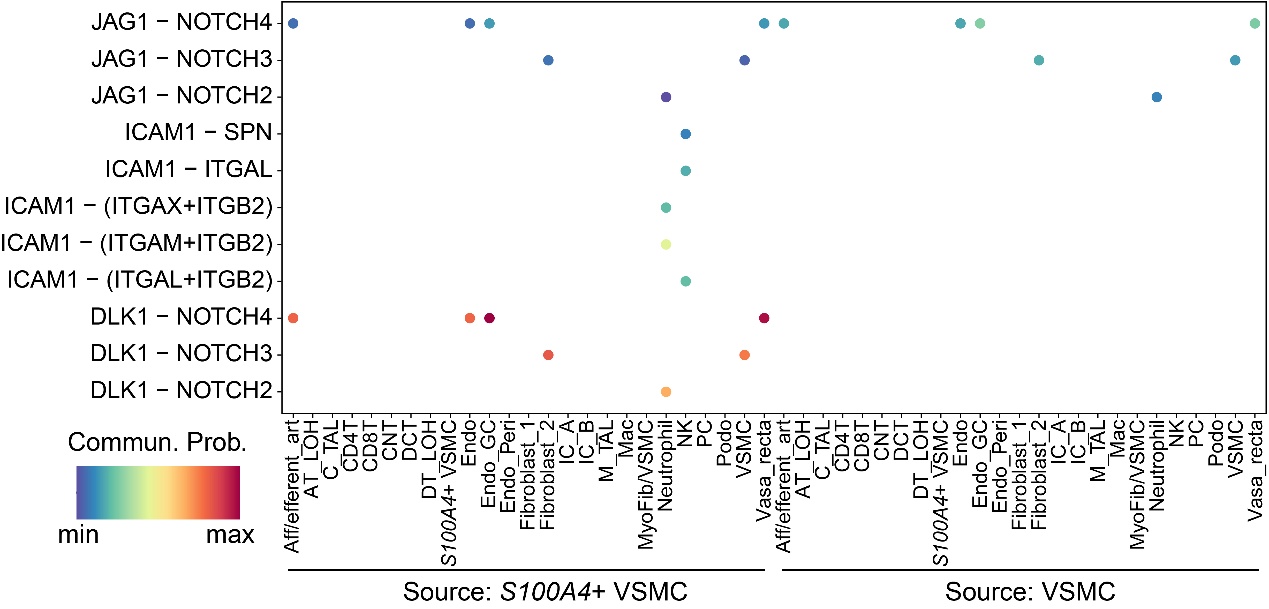


**Supplementary Fig. 8:** CellChat-inferred cell-cell contact interactions between *S100A4*⁺ VSMCs or contractile VSMCs as senders and other cell types as signal receivers.


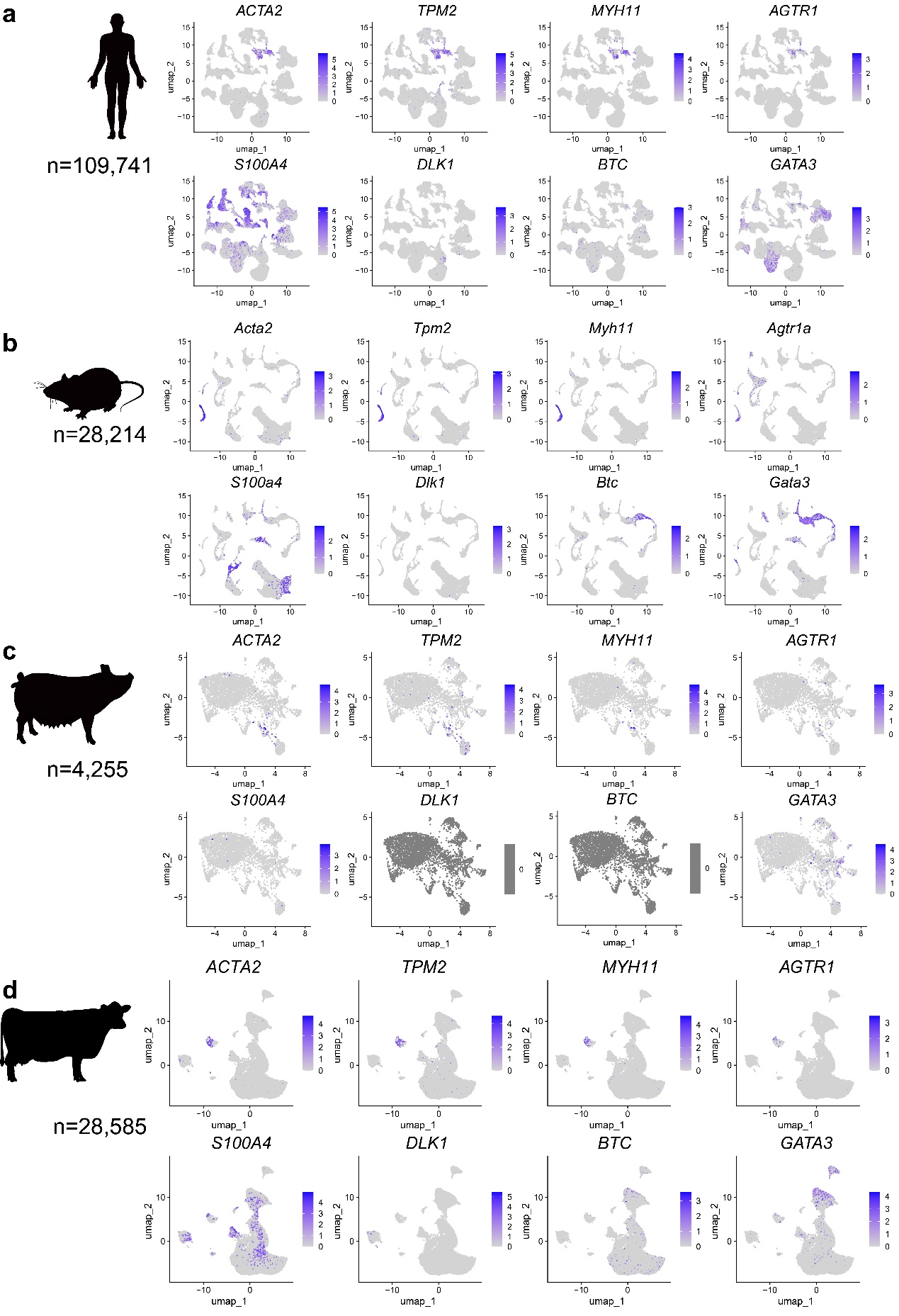


**Supplementary Fig. 9:** **The uniqueness of *S100A4*^+^ VSMCs in the camel kidney. a-d**, The expression levels of markers *ACTA2*, *TPM2*, and *MYH11* for contractile VSMCs, as well as of markers *AGTR1*, *S100A4*, *DLK1*, *BTC*, and *GATA3* for *S100A4*^+^ VSMCs in the single-cell transcriptomic atlas of human (n = 109,741) (**a**), murine (n = 28,214) (**b**), porcine (n = 4,255) (**c**), and bovine (n = 28,585) (**d**) kidneys.


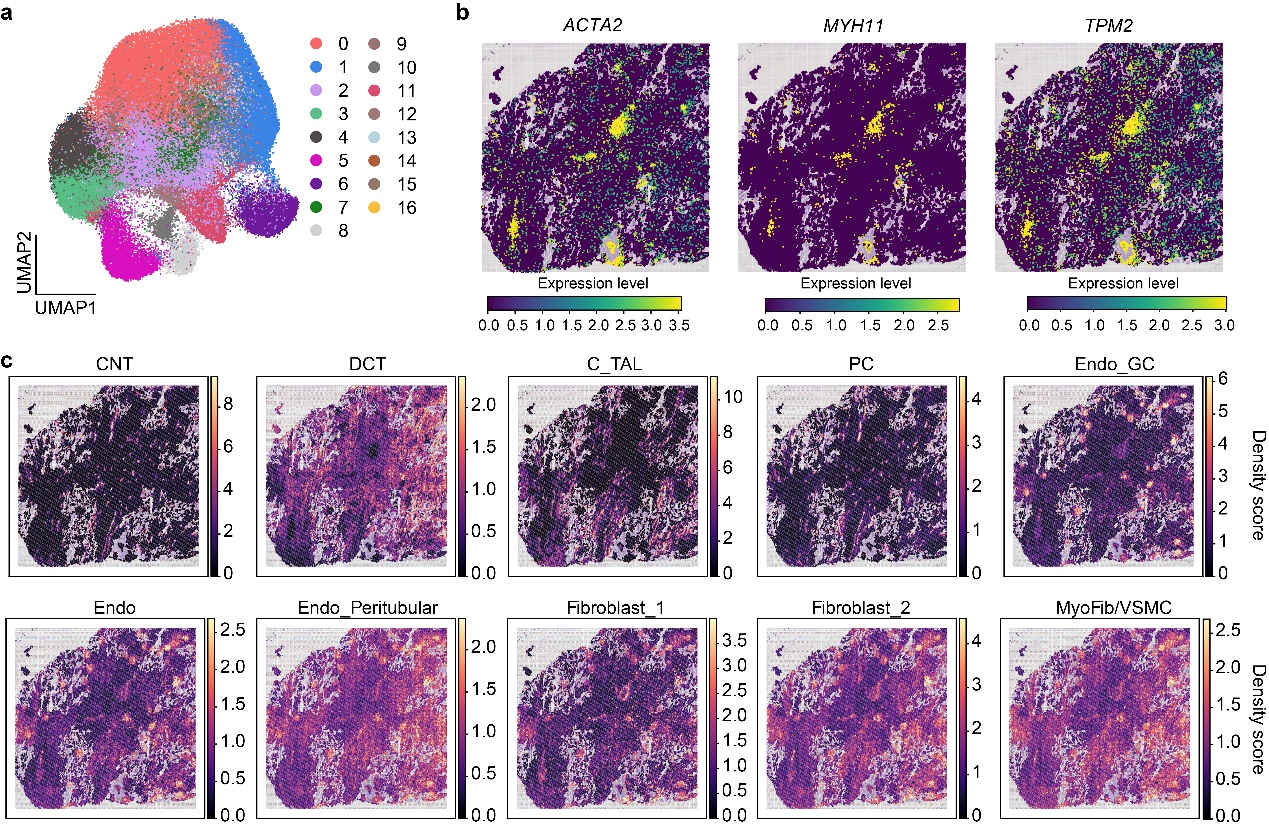


**Supplementary Fig. 10:** **The spatial localization of each cell type in the camel kidney. a**, An UMAP plot of spatial transcriptomic data from 104,965 spots. **b**, The expression levels of markers *ACTA2*, *TPM2*, and *MYH11* for contractile VSMCs. **c**, Cell2location mapping of camel renal cortex sections reveals the spatial matching of annotated cell types to their expected anatomical structures. Hematoxylin-eosin (H&E) staining images are overlaid with cell abundance density scores generated by cell2location, showing the distribution patterns of CNT, DCT, C-TAL, PC, Endo_GC, Endo, Endo_peritubular, Fibroblast_1, Fibroblast_2, and MyoFib/VSMC cell types.
